# Pretreatment ET radiomics based on primary-tumor HR status for assessing the risk of local progression after GKRS in BCBM

**DOI:** 10.3389/fonc.2026.1948516

**Published:** 2026-11-10

**Authors:** Sijun Diao, Tao Huang, Zhongyu Li, Shihong Zhu, Fujun Liu, Xiyue Lu, Huiyang Luo, Jing Chen

**Affiliations:** 1 Department of Neurosurgery, West China Hospital, West China Medical School, Sichuan University, Chengdu, China; 2 Department of Anesthesiology, West China Hospital, West China Medical School, Sichuan University, Chengdu, China

**Keywords:** breast cancer brain metastases, gamma knife radiosurgery, hormone receptor, local progression, magnetic resonance imaging, nested cross-validation, radiomics

## Abstract

**Background:**

To determine whether hormone receptor (HR) status modifies the association between pretreatment enhancing-tumor (ET) MRI radiomics and 12-month lesion-level local progression after Gamma Knife radiosurgery (GKRS) for breast cancer brain metastases (BCBM).

**Methods:**

We screened 61 patients and locked a cohort of 49 patients with 105 lesions, including 33 progressed and 72 non-progressed lesions. Enhancing tumor (ET) was manually delineated on pretreatment contrast-enhanced T1-weighted MRI. The prespecified model combined ET Rad-score, hormone receptor (HR) status and the ET Rad-score × HR interaction. Performance was evaluated using patient-grouped repeated nested cross-validation.

**Results:**

The locked model achieved lesion-level ROC-AUC 0.750, average precision (AP) 0.567 and Brier score 0.204. Patient-balanced evaluation yielded ROC-AUC 0.786, AP 0.621 and Brier score 0.194. The ET Rad-score × HR interaction was positive in 98 of 100 outer folds. HR-positive lesions had higher ROC-AUC and AP and a lower Brier score (ROC-AUC 0.827, AP 0.664, Brier score 0.174). Patient-level maximum predicted risk achieved ROC-AUC 0.803 and AP 0.694. Descriptive median-risk stratification separated lesions with progression rates of 50.9% versus 11.5%.

**Conclusion:**

Pretreatment ET radiomics showed an HR-dependent association with 12-month lesion-level local progression after GKRS in BCBM, supporting further investigation of HR-informed radiomic risk stratification. Public datasets provided construct support; endpoint-matched external validation is needed before clinical application.

## 1 Introduction

Brain metastases remain a major barrier to durable disease control in advanced breast cancer. Their risk is strongly subtype dependent, with particularly high intracranial involvement reported in HER2-positive and triple-negative metastatic breast cancer (1, 2). Stereotactic radiosurgery, including Gamma Knife radiosurgery (GKRS), is guideline-supported for selected patients with limited brain metastases and can reduce neurocognitive toxicity compared with whole-brain radiotherapy in appropriate settings (3–5). Nevertheless, local failure after SRS/GKRS remains clinically relevant in breast cancer brain metastases (BCBM). Recent breast-cancer-specific SRS data reported a 1-year local recurrence rate of approximately 29%, and a GKRS cohort also showed substantial lesion-level recurrence, especially in larger treated tumors (6, 7).

Radiomics can convert routine MRI into quantitative descriptors of tumor intensity, morphology and spatial heterogeneity (8). Prior brain-metastasis SRS/GKRS studies showed that pretreatment MRI radiomics may predict local control or recurrence, but most models treated clinical, dosimetric and molecular variables as additive predictors (9–11). In BCBM, this may be insufficient because hormone receptor (HR) status is a major biological and prognostic axis. Breast-GPA studies and receptor-discordance analyses indicate that ER/PR-defined biology influences intracranial disease behavior, and BCBM radiogenomic work suggests that MRI phenotype is associated with receptor status (12–15). Therefore, the same enhancing-tumor radiomic pattern may not carry the same risk across HR-defined contexts. This issue is particularly relevant after GKRS because local progression is assessed at the lesion level, whereas receptor status is usually abstracted at the patient or tumor-biology level. A model that explicitly tests interaction can therefore ask whether biological context changes the meaning of an imaging signal, rather than merely asking whether both variables are independently predictive.

The imaging compartment and endpoint definition were central to this study. Whole-tumor masks may mix enhancing tumor with necrotic, cystic or non-enhancing components, whereas the enhancing-tumor (ET) compartment corresponds most closely to the solid target used in radiosurgical planning (16, 17). Volumetric response assessment may also better capture small or irregular post-SRS lesions than one-dimensional criteria, although post-treatment enlargement may reflect tumor progression, radiation necrosis or mixed treatment effect (16, 18, 19). The aim of this study was to determine whether hormone receptor (HR) status modifies the association between pretreatment enhancing-tumor (ET) MRI radiomics and 12-month lesion-level local progression after Gamma Knife radiosurgery (GKRS) for breast cancer brain metastases (BCBM).

## 2 Materials and methods

### 2.1 Study design and cohort assembly

This single-center retrospective study involving human data was approved by the Ethics Committee of West China Hospital, Sichuan University (approval No. 2026 Review No. 178). The Ethics Committee waived the requirement for written informed consent for participation in this retrospective study using de-identified clinical and imaging data. Identifying fields were removed from the imaging copies used for analysis. The linkage between patient names and study identifiers was stored separately, and all images presented in the manuscript were free of identifying information. The privacy rights of all participants were observed. The study was conducted in accordance with the Declaration of Helsinki and institutional regulations.

Clinical, treatment and imaging data were assembled for 61 patients with BCBM and 159 lesion records. Eligibility required pathologically confirmed breast cancer, radiologically confirmed brain metastasis, GKRS at our center, pretreatment contrast-enhanced T1-weighted MRI, manually delineated ET ROI, matched lesion-level treatment information and follow-up sufficient for 12-month lesion-level local progression assessment. Pretreatment whole-tumor (WT) volume was required to be between 0.064 and 10 cm³, inclusive. Fifty-four lesions were excluded: nine because of adjudicated radiation necrosis, 24 because the patients died before 12 months without an available 12-month MRI assessment, one because of incomplete imaging, and 20 solely because pretreatment WT volume fell outside the eligible range. Twelve patients had no eligible lesions remaining. The cohort flow and mutually exclusive primary exclusion reasons are detailed in **Supplementary Table S1**. After imaging, ROI, clinical and endpoint curation, the locked cohort comprised 49 patients and 105 lesions, including 33 progressed and 72 non-progressed lesions. Lesions were the unit of prediction. All development, validation, tuning and sensitivity-/specificity-targeted threshold-selection procedures were grouped by patient identifier to prevent lesions from the same patient entering both training and test folds.

HR status was determined from the initial immunohistochemical assessment of the primary breast tumor at the Department of Pathology, West China Hospital; intracranial receptor reassessment was not performed as part of this study. HR-positive disease was defined as estrogen receptor and/or progesterone receptor positivity; HR-negative disease was defined as both markers negative. HR status was available for 48 of 49 patients. One HR-unknown patient contributed three lesions and was handled within the modeling pipeline by training-fold median imputation. Cohort flow, endpoint locking and HR-status completeness are further described in **Supplementary Methods 1** and **Supplementary Table S1**.

### 2.2 GKRS treatment, treatment-file audit and dose variables

All treatments were delivered using Gamma Knife Icon with Leksell GammaPlan version 11.1.1. Pretreatment contrast-enhanced T1-weighted MRI was the principal planning and radiomic sequence; T2-weighted and/or FLAIR images were used as supportive sequences for boundary assessment, edema evaluation and treatment planning when available. GKRS targets were delineated on the planning MRI by an experienced Gamma Knife neurosurgeon and reviewed by the institutional GKRS team before irradiation. Plan review followed institutional practice and included assessment of target coverage, isodose distribution and normal-tissue safety.

Treatment-session number, session-specific prescription or margin dose, prescription isodose line, target volume and dose-derived variables were extracted from treatment files when available. In the locked 105-lesion cohort, 76 lesions were treated with single-session GKRS and 29 lesions were recorded as planned non-single-session treatment. Median first-session prescription or margin dose, prescription isodose line and equivalent single-fraction dose are reported in **Table 1**. Full dose-variable definitions and treatment-file audit procedures are provided in **Supplementary Methods 2**.

**Table 1:** Baseline patient-level, lesion-level and GKRS treatment characteristics.

Characteristic	Overall	Progressed	Non-progressed
Patient-level characteristics			
Patients, n	49	22	27
Age at GKRS, years	51.0 (43.0-59.0)	50.5 (43.2-59.0)	51.0 (42.0-57.5)
KPS score	90 (90–100)	90 (82–100)	90 (90–100)
Number of brain metastases before first GKRS	2 (1-4)	2 (1-4)	2 (2-4)
HR-positive	18/48 (37.5%)	8/21 (38.1%)	10/27 (37.0%)
HR-negative	30/48 (62.5%)	13/21 (61.9%)	17/27 (63.0%)
HER2 IHC 3+	29/48 (60.4%)	15/21 (71.4%)	14/27 (51.9%)
Recorded subtype: HER2-positive	26/49 (53.1%)	10/22 (45.5%)	16/27 (59.3%)
Recorded subtype: Luminal B	17/49 (34.7%)	8/22 (36.4%)	9/27 (33.3%)
Triple-negative breast cancer	6/49 (12.2%)	4/22 (18.2%)	2/27 (7.4%)
Extracranial metastasis	34/49 (69.4%)	14/22 (63.6%)	20/27 (74.1%)
Prior WBRT before GKRS	12/49 (24.5%)	8/22 (36.4%)	4/27 (14.8%)
Prior craniotomy before GKRS	13/49 (26.5%)	10/22 (45.5%)	3/27 (11.1%)
Lesion-level imaging characteristics			
Lesions, n	105	33	72
ET volume, cm³	0.46 (0.19-1.30)	1.04 (0.30-2.28)	0.36 (0.18-0.90)
WT volume, cm³	0.46 (0.19-1.42)	1.19 (0.33-2.28)	0.37 (0.18-1.13)
NET present	51/105 (48.6%)	17/33 (51.5%)	34/72 (47.2%)
FLAIR/edema ROI available	66/105 (62.9%)	20/33 (60.6%)	46/72 (63.9%)
GKRS treatment characteristics, lesion level			
Single-session GKRS	76/105 (72.4%)	21/33 (63.6%)	55/72 (76.4%)
Planned non-single-session GKRS	29/105 (27.6%)	12/33 (36.4%)	17/72 (23.6%)
First-session prescription/margin dose, Gy	13.0 (12.0-18.0)	13.0 (12.0-18.0)	13.0 (12.0-18.0)
First-session prescription isodose line, %	45.0 (45.0-50.0)	45.0 (45.0-50.0)	45.0 (45.0-50.0)
Second-session prescription/margin dose, Gy	13.0 (12.0-13.0)	12.0 (12.0-13.0)	13.0 (13.0-13.0)
Second-session prescription isodose line, %	45.0 (45.0-50.0)	45.0 (45.0-50.0)	45.0 (45.0-45.0)
Equivalent single-fraction dose, α/β = 10 Gy	18.0 (12.0-20.0)	18.0 (14.0-18.5)	17.8 (12.0-20.0)
Same-course treated lesion count	4 (2-4)	3 (1-4)	4 (2-4)
Same-course cumulative treated volume, cm³	1.92 (1.16-5.27)	4.43 (2.02-6.25)	1.35 (0.91-2.40)
Dose features usable	103/105 (98.1%)	32/33 (97.0%)	71/72 (98.6%)

Values are median (interquartile range) or n (%). Patient-level groups are based on whether a patient had any progressed lesion; lesion-level rows refer to the lesion-level endpoint. HR-positive was ER-positive and/or PR-positive. Second-session summaries include planned non-single-session lesions only. Equivalent single-fraction dose used α/β = 10 Gy. ET, enhancing tumor; FLAIR, fluid-attenuated inversion recovery; GKRS, Gamma Knife radiosurgery; HR, hormone receptor; IHC, immunohistochemistry; KPS, Karnofsky Performance Status; NET, non-enhancing tumor; ROI, region of interest; WBRT, whole-brain radiotherapy; WT, whole tumor.

Derived tumor-dose summaries used the linear-quadratic model to calculate BED10 and an equivalent single-fraction dose for exploratory analyses (20); formulas and assumptions are provided in **Supplementary Methods 2**. Planned non-single-session GKRS was handled as one index course, assuming sufficient repair between sessions (21).

RTSTRUCT, RTPLAN and RTDOSE files were audited for readability, frame-of-reference consistency and lesion-to-target correspondence; full audit counts are provided in **Supplementary Methods 2** and **Supplementary Table S2**. Normal-tissue constraints were reviewed clinically but were not modeled as locked predictors because consistently extractable critical-structure dose variables were unavailable for all lesions. Treatment-file and dose-audit results documented treatment quality and defined exploratory dosimetric analyses; they were not used to define the locked primary ET-HR model. Repeat or salvage GKRS was adjudicated against the index lesion: baseline features came only from the index pretreatment MRI, progression before salvage was counted at the first qualifying MRI or salvage date when the MRI date was unavailable, and new metastases after index GKRS were considered distant intracranial events rather than local failure of the original lesion.

Systemic therapy was abstracted for the locked 49-patient cohort. Drug names, start and end dates, additions, switches and confirmed treatment-free intervals were reconciled. The treatment inventory covered index GKRS through its calendar 12-month anniversary, including the overlapping exposure of regimens begun before GKRS. Treatments were summarized once per patient within each category or regimen for patients with any-lesion progression (n = 22) versus none (n = 27). Treatment active at GKRS was distinguished from subsequent exposure; whole-window use could include post-progression treatment. Major treatment categories summarized in the main text included HER2-targeted antibody–drug conjugates, HER2 tyrosine kinase inhibitors, conventional chemotherapy and endocrine therapy; detailed categories and individual regimens are provided in **Supplementary Methods 7** and **Supplementary Tables S13**-**S14**.

### 2.3 MRI acquisition, ROI delineation and feature extraction

Pretreatment MRI was acquired on a 3.0-T GE SIGNA Premier scanner. Contrast-enhanced T1-weighted MRI used gadopentetate dimeglumine 0.1 mmol/kg and 1.4-mm slice thickness. T2-FLAIR imaging used 3-mm slice thickness and served as supportive information when available. MRI data were converted from DICOM to NRRD and processed using N4 bias-field correction, 1-mm isotropic resampling and intensity standardization before radiomic extraction.

Two neurosurgeons independently delineated the lesions slice by slice on pretreatment contrast-enhanced T1-weighted MRI in 3D Slicer. ET was defined as enhancing solid tumor; visually non-enhancing, necrotic, cystic or hemorrhagic components were excluded when present. Reader 1 was an associate chief neurosurgeon with 8 years of experience in neuro-oncologic imaging and GKRS, and Reader 2 was an attending neurosurgeon with 7 years of experience. A senior chief neurosurgeon with 25 years of experience reviewed both sets of segmentations and adjudicated discrepancies. If disagreement persisted, the three neurosurgeons discussed the case and selected the final segmentation by majority opinion. The final adjudicated segmentation was used for the primary model; Dice and ICC analyses used the two independent reader-specific sets before adjudication (**Supplementary Methods 5**). The readers were blinded to local failure, survival, molecular subtype, HR/HER2 status and radiomic features. Radiomic features were extracted from the locked ET ROI using PyRadiomics. The broader extraction framework included Original, Wavelet and Laplacian-of-Gaussian image types; the locked primary model used 93 Original first-order and texture features, excluding shape features. Whole-tumor (WT), non-enhancing tumor (NET), edema and Ring3 features were evaluated only as exploratory model-boundary analyses. Detailed radiomics extraction and modelling settings are provided in **Supplementary Methods 3** and **Supplementary Table S3**.

Inter-reader reproducibility was evaluated using the two reader-specific segmentation sets for all 105 lesions in the locked cohort. Spatial agreement was summarized using Dice similarity. Absolute-agreement, single-measurement ICCs were calculated for the 93 candidate ET features, with 95% intervals from 5, 000 patient-cluster bootstrap resamples. This analysis was not part of the original feature-selection pipeline, and no ICC-based filtering threshold was applied during original model development. Detailed methods and results are provided in **Supplementary Methods 5** and **Supplementary Tables S9**-**S10**.

### 2.4 Endpoint definition and ET-HR modeling

The primary endpoint was local progression within 12 months after index GKRS. Lesions were counted as events if local progression was documented within 12 months; lesions without progression were considered non-progressed only if they had at least 12 months of lesion-level follow-up. Lesions with unresolved progression versus treatment-effect ambiguity, missing endpoint information or insufficient follow-up were excluded before model locking. Although RANO-BM provides the commonly used one-dimensional framework for brain metastasis response assessment, the present lesion-level analysis used the total-volume volumetric criteria proposed by Ocaña-Tienda et al., which were developed and benchmarked against RANO-BM to address limitations of one-dimensional assessment in brain metastases. Partial response was defined as at least a 20% volume reduction relative to baseline (16, 19). Progressive disease was defined as at least a 30% volume increase relative to the prior minimum volume, including baseline; when the prior minimum volume was below 1 cm³, progressive disease additionally required an absolute volume increase of at least 0.2 cm³. Complete response was defined as disappearance of the lesion sustained for at least four weeks, and all other patterns were classified as stable disease.

Follow-up MRI was assessed by an experienced institutional radiologist through longitudinal comparison of pretreatment images and scans obtained at approximately 3, 6 and 12 months after GKRS. Enlargement on a single scan was not sufficient to establish tumor progression. Lesions with early enlargement at approximately 3 months and suspected treatment-related changes were reviewed using subsequent MRI evolution and intervening treatment records. Subsequent regression without repeat local irradiation or a change in systemic anticancer treatment supported a treatment-related explanation, but was not regarded as pathological confirmation of radiation necrosis. These cases underwent review and final adjudication by a second experienced neurosurgeon. The actual evidence used consisted of serial conventional MRI and clinical treatment records. Outcome adjudication and eligibility decisions preceded model development, so assessors could not access ET Rad-scores or model predictions. Nine lesions adjudicated as radiological radiation necrosis were excluded before model development (**Supplementary Methods 1**; **Supplementary Table S1**).

High-dimensional ET features were converted into a fold-specific ET Rad-score inside the training data of each resampling split. Features with excessive missingness, near-zero variance or high redundancy were removed; candidates were ranked by absolute Spearman correlation with the training outcome, standardized and fitted using ridge logistic regression with balanced class weights. When more than five features remained, the five largest absolute training-fold coefficients were retained. The Rad-score was calculated as the fitted intercept plus the weighted sum of the retained standardized features; it was a continuous score, not a predicted probability. The locked primary model included the ET Rad-score, primary-tumor HR status (0 for HR-negative and 1 for HR-positive) and their product, each standardized using the training data. This interaction term allowed the association between the Rad-score and the log odds of local progression to differ by HR status. Calculation details are provided in **Supplementary Methods 6**. Feature-selection stability was summarized across the 100 outer training folds by recording how often each feature was retained and the sign of its coefficient when selected. Selection frequency was calculated as the number of folds retaining a feature divided by 100. The final fitted ET Rad-score comprised firstorder_Maximum, glcm_Idmn, glrlm_RunVariance, glszm_GrayLevelVariance and glszm_LargeAreaHighGrayLevelEmphasis. Their selection frequencies were 68%, 16%, 28%, 74% and 52%, respectively, and their coefficients were positive whenever selected. Feature-level counts and coefficient summaries are provided in **Supplementary Table S4**.

### 2.5 Statistical analysis

Analyses were performed in Python. Model performance was estimated using patient-grouped repeated nested cross-validation with 20 repeats, five outer folds and four inner folds. Imputation, standardization, feature selection, ET Rad-score construction, hyperparameter tuning, calibrator fitting and sensitivity-/specificity-targeted threshold selection were performed within training folds, with the fitted transformations, models and thresholds applied to held-out outer-fold lesions. Primary performance estimates and calibration plots were based on each lesion’s mean probability across 20 held-out outer-fold predictions, without recalibration. Calibration slope and intercept were estimated from these mean probabilities to assess calibration. For sensitivity- and specificity-targeted strategies, each lesion was classified using the corresponding training-fold threshold in each repeat. At least 11 of 20 positive classifications defined high risk; a 10:10 tie was assigned to low risk. Descriptive median-risk stratification instead used the overall median of the 105 lesion-level mean OOF probabilities (0.43956342220446454), with values at or above the cutoff assigned to high risk and lower values to low risk. This overall-median split was used for descriptive stratification rather than as a training-fold-selected operating threshold. Details of calibrator fitting and threshold selection are provided in **Supplementary Methods 6**.

Discrimination was assessed using ROC-AUC and average precision (AP), where AP represents average precision rather than trapezoidal precision-recall AUC (22, 23). Probabilistic accuracy was assessed using Brier score (23, 24). Primary 95% confidence intervals used patient-clustered bootstrap resampling with 1, 500 resamples. Patient-balanced and patient-level confidence intervals used patient-level clustered bootstrap resampling with 5, 000 resamples. These intervals account for within-patient lesion clustering but do not incorporate uncertainty from repeated retraining of the full analytical pipeline. Patient-level performance used maximum predicted lesion risk for any-lesion progression, with mean and ET-volume-weighted mean risk as sensitivity summaries. Operating points were summarized using sensitivity, specificity, predictive values, risk-group fractions and progression-capture counts. For descriptive median-risk stratification, the odds ratio (OR) and log-Wald 95% confidence interval were calculated after adding 0.5 to each cell of the 2 × 2 table, without covariate adjustment; this interval did not account for within-patient lesion clustering. Public datasets were interpreted as construct-level, endpoint-adjacent or technical support rather than endpoint-matched external validation.

HR-stratified 95% confidence intervals were obtained from 5, 000 patient-cluster bootstrap resamples within each HR group, retaining all lesions from each sampled patient and holding the existing out-of-fold predictions fixed. The interaction coefficient distribution across 100 correlated outer folds was summarized descriptively and was not interpreted as a population-effect confidence interval (**Supplementary Tables S11**, **S12**).

Sensitivity analyses added baseline log-transformed ET volume, prior whole-brain radiotherapy (WBRT), prior craniotomy, equivalent single-fraction dose, baseline HER2-TKI use or baseline HER2-ADC use to the locked ET-HR model, one factor at a time. We retained the original patient-grouped outer splits and each fold’s original inner-selected regularization parameter, while refitting the training-fold Rad-score and extended model. Performance differences used 5, 000 paired patient-cluster bootstrap resamples of averaged out-of-fold predictions (**Supplementary Methods 8**; **Supplementary Tables S15**–**S17**).

## 3 Results

### 3.1 Cohort, baseline and treatment characteristics

The locked cohort included 49 patients with 105 lesions; 33 lesions had 12-month local progression and 72 did not (**Figure 1**). Median age was 51.0 years, median KPS was 90 and the median number of brain metastases before first GKRS was 2. HR status was known in 48 patients: 18 were HR-positive and 30 were HR-negative. Progressed lesions were descriptively larger than non-progressed lesions, with median ET volume 1.04 versus 0.36 cm³. GKRS treatment variables are summarized in **Table 1**. Planned non-single-session treatment was more frequent among progressed lesions, whereas dose features were usable in 103 of 105 lesions.

**Figure 1:**
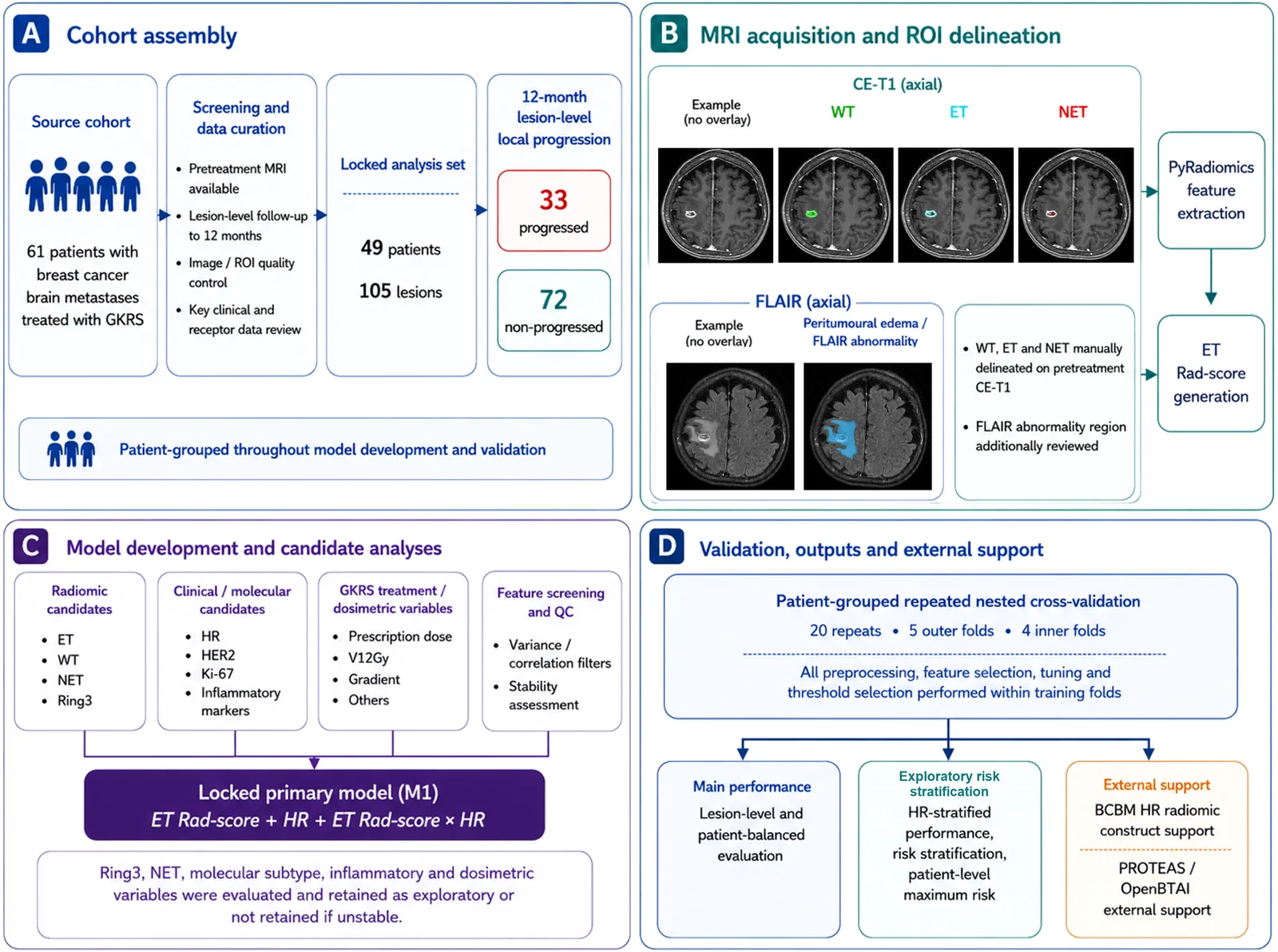
Study workflow and manual ROI framework. **(A)** Cohort assembly: 61 patients were screened, and 49 patients with 105 lesions were included; 33 lesions progressed and 72 did not. **(B)** Manual delineation of whole tumor (WT) and enhancing tumor (ET) on pretreatment contrast-enhanced T1-weighted MRI. Non-enhancing tumor (NET) and FLAIR abnormality were exploratory or supportive compartments. **(C)** Candidate analyses and the locked primary model (M1), comprising ET Rad-score, HR status and their interaction. **(D)** Patient-grouped repeated nested cross-validation (20 repeats, 5 outer folds and 4 inner folds), model outputs and public-data support. Preprocessing, feature selection, tuning and targeted threshold selection were confined to training folds.

During the 12-month post-GKRS window, HER2-targeted antibody–drug conjugates were recorded in 4/22 patients with any-lesion progression and 10/27 without progression; HER2 tyrosine kinase inhibitors in 11/22 and 14/27; conventional chemotherapy in 19/22 and 20/27; and endocrine therapy in 2/22 and 5/27, respectively. Treatment categories overlapped because patients could receive combined or sequential regimens (**Supplementary Tables S13**, **S14**).

Median inter-reader Dice similarity for ET segmentation was 0.937 (IQR, 0.894–0.958). Across the 93 ET radiomic features, median inter-reader ICC was 0.928 (range, 0.757–0.999), with 62 features having ICC estimates ≥0.900. The five features included in the final ET Rad-score had ICCs ranging from 0.890 to 0.992; the lower bounds of their patient-cluster bootstrap 95% confidence intervals ranged from 0.812 to 0.973 (**Supplementary Tables S9**, **S10**).

### 3.2 Locked ET-HR model performance

The ET-HR interaction model achieved lesion-level ROC-AUC 0.750 (95% CI, 0.642-0.851), AP 0.567 (95% CI, 0.402-0.768) and Brier score 0.204 (95% CI, 0.177-0.235) (**Figure 2**; **Supplementary Table S5**). Patient-balanced evaluation yielded ROC-AUC 0.786, AP 0.621 and Brier score 0.194. When lesion risks were aggregated to the patient level, maximum predicted lesion risk achieved ROC-AUC 0.803, AP 0.694 and Brier score 0.200 for any-lesion progression.

**Figure 2:**
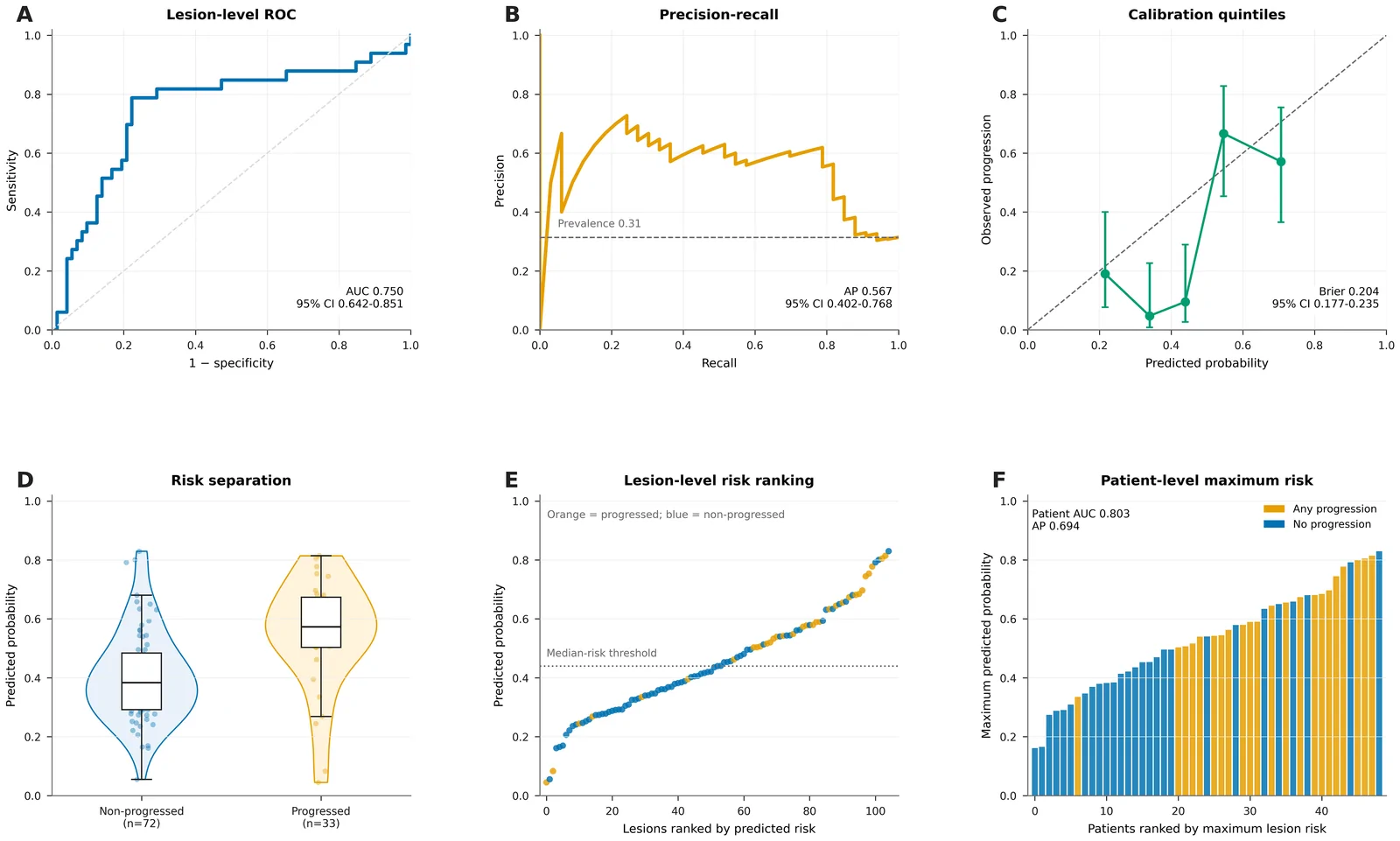
Main model performance and calibration. **(A)** Lesion-level receiver operating characteristic curve for the locked ET-HR interaction model. **(B)** Precision–recall curve for lesion-level 12-month local progression, reported with average precision (AP). **(C)** Calibration across predicted-risk quintiles, comparing predicted probability with observed progression. **(D)** Distribution of predicted probabilities in progressed and non-progressed lesions. **(E)** Lesion-level risk ranking based on out-of-fold predicted probabilities; the dotted horizontal line indicates the median-risk threshold used for risk stratification. **(F)** Patient-level aggregation using maximum predicted lesion risk per patient for any-lesion progression. Panels A-E use the mean of 20 raw outer-test probabilities per lesion; panel F takes the maximum of these lesion-level means within each patient. The calibration curve is an evaluation, not a recalibration of the primary predictions.

Excluding the single HR-unknown patient after prediction aggregation produced similar performance in the HR-known restricted set (48 patients/102 lesions: ROC-AUC 0.755, AP 0.573 and Brier score 0.202, **Supplementary Table S6**).

### 3.3 HR-associated variation in ET radiomic risk

The standardized coefficient of the ET Rad-score × HR product term was positive in 98 of 100 outer validation folds, with a median of 0.326 (IQR, 0.207–0.445; range, −0.087 to 1.154; **Figure 3**; **Supplementary Table S12**). HR-positive lesions had higher ROC-AUC and AP and a lower Brier score than HR-negative lesions: ROC-AUC 0.827, AP 0.664 and Brier score 0.174 versus ROC-AUC 0.702, AP 0.562 and Brier score 0.215. The HR-positive subgroup included 18 patients, 32 lesions and 11 events; the HR-negative subgroup included 30 patients, 70 lesions and 21 events. Patient-cluster bootstrap ROC-AUC intervals were 0.614-1.000 and 0.554-0.831, respectively; AP and Brier intervals are reported in **Supplementary Table S11**. Within HR-positive lesions, ET Rad-score alone reached AUC 0.835 and GrayLevelVariance reached AUC 0.827. BCBM-RadioGenomics showed concordant HR-related ET feature differences as construct support, not endpoint validation.

**Figure 3:**
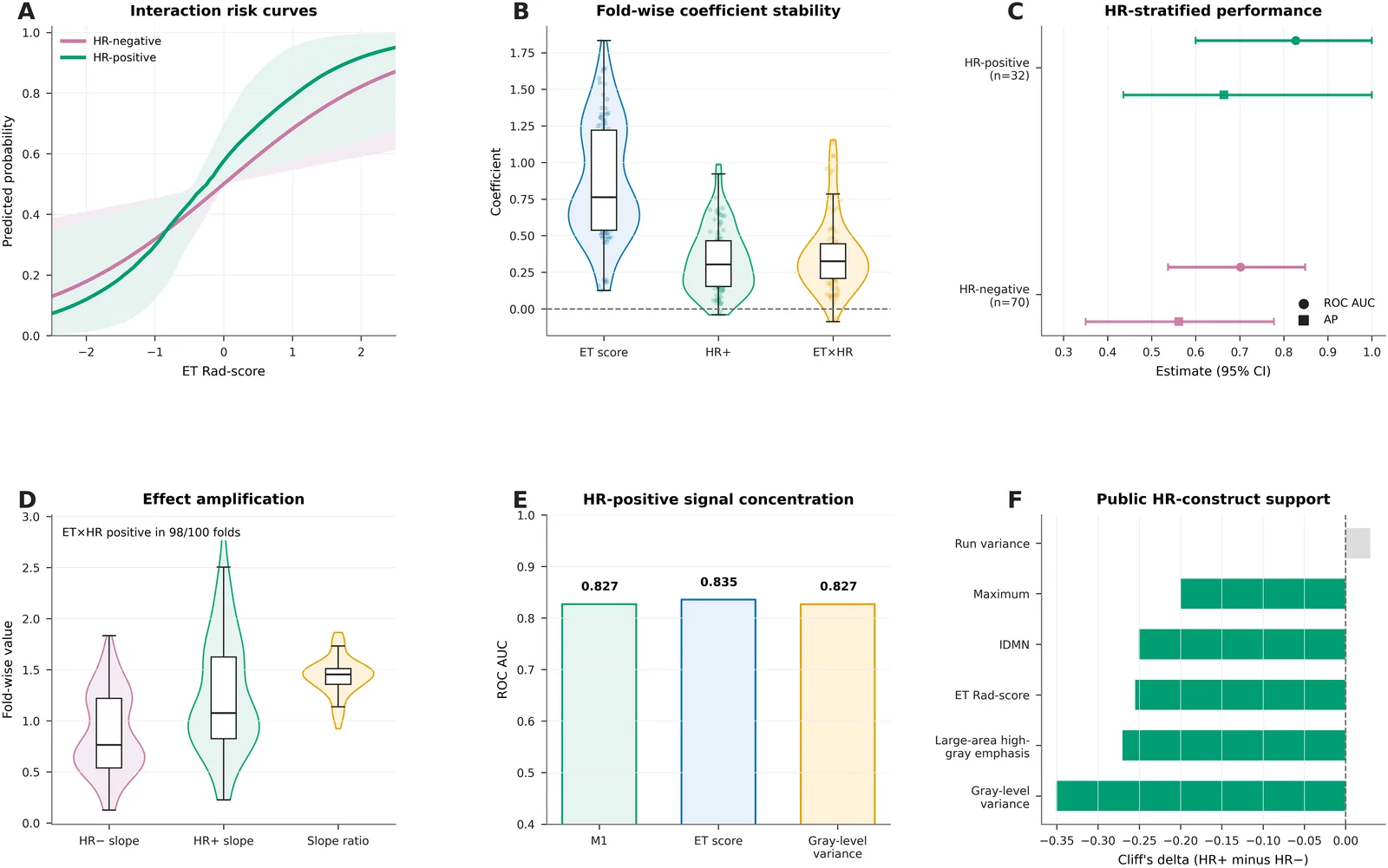
Hormone receptor status modifies ET radiomic risk. **(A)** Predicted 12-month local progression probability versus ET Rad-score by HR status. **(B)** Standardized coefficient distributions across outer validation folds. These repeated folds are correlated, not independent observations. **(C)** HR-stratified model performance; detailed estimates and patient-cluster confidence intervals are reported in **Supplementary Table S11**. **(D)** Fold-specific ET Rad-score slopes by HR status. **(E)** Primary-model and exploratory single-marker discrimination in HR-positive lesions. **(F)** HR-related ET feature differences in the public BCBM dataset, expressed as Cliff’s delta for HR-positive minus HR-negative lesions, providing construct support rather than endpoint validation. Interaction coefficient summaries are provided in **Supplementary Table S12**.

### 3.4 Targeted sensitivity analyses

Across six sensitivity analyses that individually added baseline ET volume, prior whole-brain radiotherapy, prior craniotomy, equivalent single-fraction dose, baseline HER2-TKI use or baseline HER2-ADC use, the ET Rad-score × HR interaction remained positive in 97–98 of 100 outer folds. Median standardized interaction coefficients ranged from 0.314 to 0.332, compared with 0.326 in the primary model. ROC-AUC ranged from 0.730 to 0.758, AP from 0.542 to 0.584 and Brier score from 0.195 to 0.210, compared with 0.750, 0.567 and 0.204, respectively, in the primary model. Predictive performance did not improve consistently across the six extensions. The extension incorporating baseline HER2-TKI use yielded lower AP and a higher Brier score than the primary model; the dose extension also yielded a slightly higher Brier score. Model-specific estimates, interaction coefficient distributions and paired performance differences with 95% confidence intervals are reported in **Supplementary Tables S15**–**S17**.

### 3.5 Clinical stratification and model boundaries

Threshold analyses yielded exploratory operating points (**Figure 4**; **Supplementary Table S7**). The 90%-target low-risk exclusion strategy classified 37.1% of lesions as low risk, with sensitivity 0.848 and NPV 0.872; five of 33 progressed lesions fell below this threshold. The 90%-target high-specificity strategy identified 23.8% as high risk, with specificity 0.875 and PPV 0.640. Progression increased across tertiles from 5/35 to 8/35 and 20/35 lesions. Descriptive median-risk stratification classified 53 lesions as high risk and 52 as low risk, with progression rates of 50.9% versus 11.5% (OR 7.42; 95% CI, 2.79-19.76). The four-cell counts and classification metrics are reported in **Supplementary Table S7**. Exploratory analyses did not support replacing the locked ET-HR model: clinical-dose modeling and WT-only radiomics were weak, Ring3 lacked robustness for locking, and HER2, Ki-67, inflammatory and dosimetric extensions did not provide stable incremental value (**Supplementary Figures S1**–**S5**). These earlier model-boundary comparisons used different specifications and are distinct from the targeted ET-HR sensitivity analyses in Section 3.4. Public datasets provided construct-level or technical support but no complete endpoint-matched external validation; dataset-specific support roles, analyses and claim boundaries are summarized in **Supplementary Table S8** (25–27).

**Figure 4:**
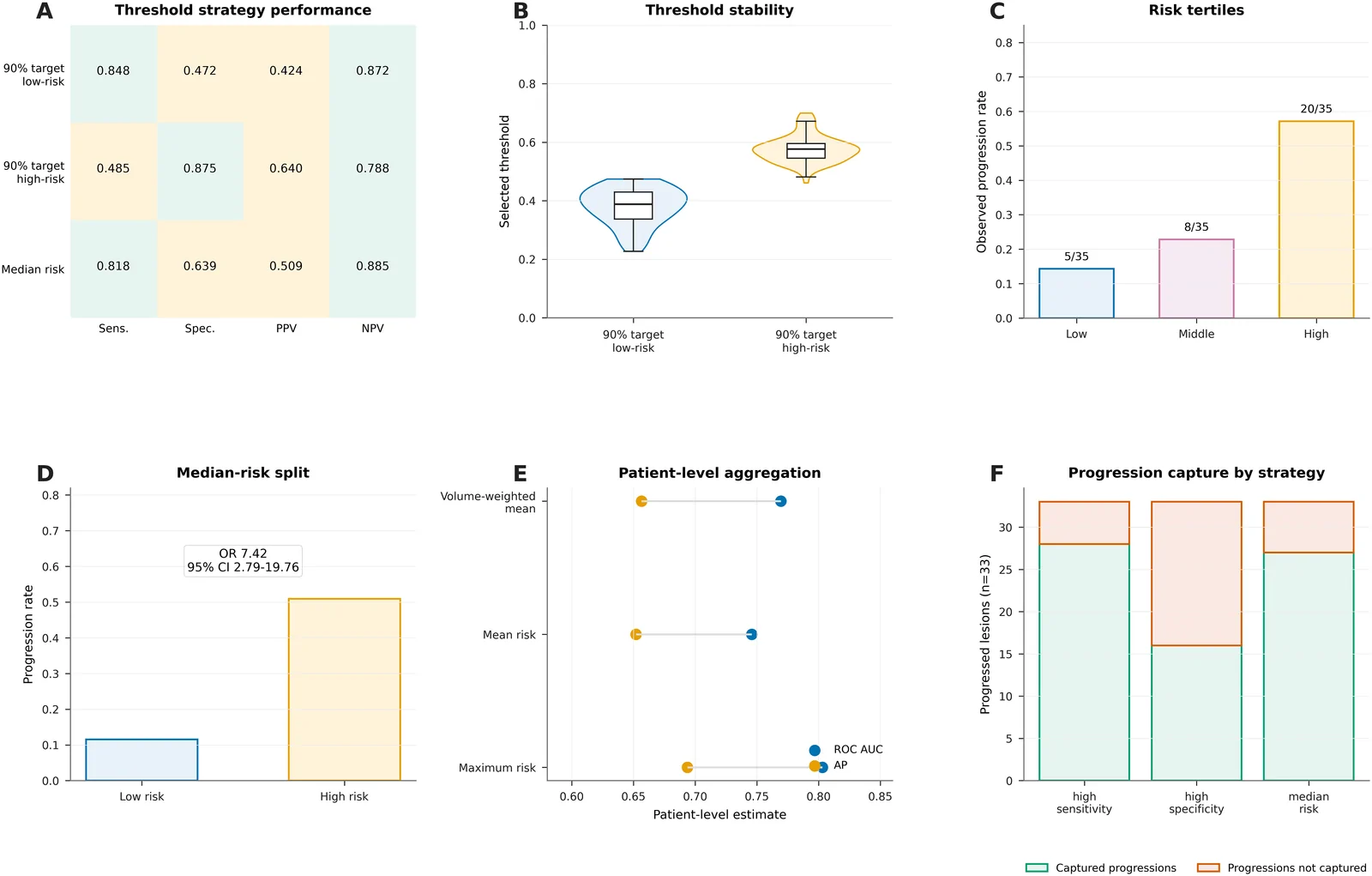
Exploratory threshold analysis and risk stratification of the ET-HR interaction model. **(A)** Classification performance. **(B)** Training-fold threshold distributions for the sensitivity- and specificity-targeted strategies. **(C)** Progression rates across predicted-risk tertiles. **(D)** Descriptive median-risk stratification. **(E)** Patient-level prediction of any-lesion progression using maximum, mean and volume-weighted mean lesion risk. **(F)** Progression capture by strategy. Targeted strategies used training-fold thresholds and majority voting across 20 held-out classifications. Descriptive median-risk groups used the overall median of lesion-level mean OOF probabilities (approximately 0.4396; unrounded cutoff used), with values at or above the cutoff classified as high risk. The OR used a 0.5 correction to all four table cells without covariate adjustment; its log-Wald 95% CI did not account for within-patient lesion clustering. **Supplementary Table S7** provides group counts, classification metrics and the 95%-sensitivity-target analysis.

## 4 Discussion

This study suggests that pretreatment ET radiomics after GKRS has HR-conditioned prognostic meaning in BCBM. The key finding is not simply that radiomics predicted 12-month local progression, but that the imaging-risk association varied with primary-tumor-defined HR status in a cohort treated with audited GKRS plans and lesion-level volumetric follow-up (16, 19). The compact ET-HR interaction model retained discrimination under patient-balanced evaluation and translated lesion-level risk into a patient-level maximum-risk summary. The positive ET Rad-score × HR interaction in 98 of 100 outer folds describes resampling stability of the interaction direction; it does not establish a causal modifying effect of HR or an independent population-level interaction test. The predominant interaction direction was retained in the targeted sensitivity analyses, although predictive performance did not consistently improve and residual confounding remains possible (**Supplementary Tables S15**–**S17**).

From a radiotherapy perspective, the study suggests that pretreatment imaging risk after GKRS should not be interpreted independently of tumor biology. Breast-GPA, subtype-switching and receptor-discordance studies indicate that receptor-defined biology has prognostic and therapeutic relevance in BCBM (12, 13, 15). BCBM radiomics studies further suggest that MRI phenotype may reflect receptor status or molecular subtype, including HR-related subtype conversion (28, 29). Subtype-specific radiosurgery cohorts in HER2-positive and triple-negative BCBM, together with studies of CNS-active HER2-targeted systemic therapy, also indicate that local post-SRS behavior may occur within different systemic-treatment and biological contexts (30–33). The same ET radiomic pattern may therefore imply different post-treatment behavior in HR-positive and HR-negative disease, which may partly explain why broad feature-fusion models are unstable in small radiosurgery cohorts. This supports a constrained modeling strategy in which radiomic signals are tested within biologically meaningful contexts rather than simply added to clinical and dosimetric covariates.

The biological basis of this interaction remains hypothesis-generating. HR-positive and HR-negative BCBM may differ in clinical course, receptor concordance or discordance, imaging phenotype, treatment exposure and radiosensitivity, all of which could change how pretreatment ET texture relates to subsequent local progression (30, 31, 34). These findings suggest that the association between ET radiomic features and local progression varies according to primary-tumor HR status.

These findings extend prior brain-metastasis SRS/GKRS radiomics studies, which mainly evaluated radiomic, clinical or dosimetric variables as additive predictors, by asking a different question: whether receptor-defined breast cancer biology changes the risk meaning of the same pretreatment imaging phenotype (9–11). The retained ET compartment is also clinically interpretable because it corresponds to the enhancing solid component most relevant to radiosurgical planning, for which high-resolution contrast-enhanced MRI remains central (35). Exploratory WT, NET, edema, Ring3 and dose-related extensions did not provide stable incremental value. This does not imply that these compartments or treatment variables are unimportant; rather, in this dataset they were not robust enough to expand the locked model without increasing complexity and instability.

The endpoint definition is also important for interpreting these results. RANO-BM remains the commonly used one-dimensional response framework for brain metastases, but diameter-based assessment can be limited for small, irregular or ring-enhancing lesions after SRS/GKRS (16). We therefore used the total-volume criteria proposed by Ocaña-Tienda et al., which were developed and benchmarked against RANO-BM and reported broader lesion evaluability, reduced misclassification and earlier response assessment (19). This approach allowed lesions progressing before 12 months to be counted as events while avoiding the classification of short-follow-up non-events as durable local control. It also aligned the endpoint with the lesion-level volumetric measurements used in post-GKRS follow-up.

The model may help identify patients for whom closer MRI surveillance warrants evaluation. Prospective studies in independent cohorts should assess whether shorter MRI follow-up intervals in the high-risk group improve timely detection of local progression and whether longer intervals in the low-risk group reduce imaging burden without delaying detection or salvage treatment. For patients with multiple lesions, the maximum lesion-level predicted risk could be evaluated as a patient-level summary to guide surveillance scheduling. Such studies should compare risk-guided surveillance with standard follow-up and assess missed or delayed progression, additional imaging, unnecessary interventions and clinical net benefit (36, 37).

The public-data analyses support an HR-associated ET radiomic construct and highlight domain compatibility issues, but they do not replace endpoint-matched external validation (17, 26, 37, 38). This distinction is important for radiomics translation, where feature definitions, segmentation, scanner domain, validation design and transparent reporting strongly influence apparent performance (39–43). By keeping the model low-dimensional and by separating primary prediction from exploratory dose, molecular and public-data analyses, the present design aimed to prioritize interpretability over maximal apparent discrimination.

Several limitations remain. This retrospective, single-center study included a modest cohort, with limited precision in HR-stratified performance estimates and heterogeneous systemic treatment exposure. HR classification relied on primary-tumor IHC at initial diagnosis. Receptor discordance between primary tumors and brain metastases may therefore affect HR grouping and the estimated ET Rad-score × HR interaction, warranting evaluation in cohorts with paired tissue assessments (12, 14, 15). Outcome adjudication relied on serial conventional MRI and treatment records, leaving potential misclassification between progression and treatment-related changes (18). Although inter-reader agreement was high overall, reproducibility across a broader range of readers and imaging platforms requires further evaluation. Standardized treatment-related toxicity grading was unavailable and was not analyzed as a formal endpoint. Finally, the model has not undergone complete endpoint-matched external validation; therefore, it should be considered a hypothesis-generating, biology-conditioned risk-stratification framework.

The interaction direction remained consistent after individual adjustment for lesion volume, prior local treatment, dose and baseline HER2-directed therapy (**Supplementary Tables S15**–**S17**). The modest sample size limited joint adjustment and the precision of treatment-specific estimates, particularly for baseline HER2-ADC use (eight patients). Systemic treatment summaries covered the full 12-month window and could include post-progression therapy; their timing relative to individual lesion progression was not incorporated into these analyses. Residual confounding from treatment changes and salvage therapy therefore remains possible.

## 5 Conclusion

Pretreatment ET radiomics provides information on the risk of local progression after GKRS in BCBM that varies with primary-tumor HR status. This finding highlights the importance of considering tumor biology when interpreting imaging-derived risk and supports evaluating radiomic markers within HR-defined contexts. Integrating ET radiomics with HR status offers a basis for further development of individualized risk assessment after GKRS. Independent external validation and prospective evaluation are needed to determine whether this approach can improve the planning of MRI surveillance and subsequent clinical management.

## Data Availability

The original contributions presented in the study are included in the article/**Supplementary Material**. Further inquiries can be directed to the corresponding author.
