# Supplementary material for: Pretreatment ET radiomics based on primary-tumor HR status for assessing the risk of local progression after GKRS in BCBM
Source: Front Oncol. 2026 Nov 10;16:1948516. doi: 10.3389/fonc.2026.1948516 (PMC13757771; doi:10.3389/fonc.2026.1948516)
Supplement: Supplementary file 1 [file SupplementaryFile1.docx]

**Supplementary material**

**Supplementary Methods**

**1. Cohort flow, endpoint lock and HR-status handling**

Cohort assembly, lesion-level eligibility and HR-status completeness are summarized in Supplementary Table S1. Clinical, imaging, treatment and follow-up data were curated before model locking. Lesions were eligible for the locked primary analysis only when pretreatment contrast-enhanced T1-weighted MRI, manually delineated enhancing-tumor (ET) ROI, matched lesion-level treatment information and sufficient follow-up for 12-month local progression assessment were available. Pretreatment whole-tumor (WT) volume was required to be between 0.064 and 10 cm³, inclusive.

Endpoint status was audited at the lesion level and frozen before model development. Lesions with documented local progression within 12 months after index Gamma Knife radiosurgery (GKRS) were counted as events. Lesions without local progression were considered non-progressed only if lesion-level follow-up reached at least 12 months. Lesions with unresolved ambiguity between local progression and treatment-related change, missing endpoint information or insufficient follow-up were excluded before model locking.

An experienced radiologist assessed longitudinal conventional MRI at approximately 3, 6 and 12 months against pretreatment imaging. Early enlargement was reviewed with later imaging and intervening treatment records. Regression without repeat local irradiation or a change in systemic anticancer therapy supported treatment-related change, not pathological proof of necrosis. A second experienced neurosurgeon reviewed these cases and made the final adjudication before model development, without access to radiomic scores or predictions. Nine lesions were adjudicated as radiological radiation necrosis and excluded. The recorded unresolved-dispute flag was zero and should not be interpreted as absence of adjudicated radiation necrosis.

HR-positive disease was defined as estrogen receptor and/or progesterone receptor positivity, and HR-negative disease was defined as both markers negative. HR-known restricted evaluation used the same locked M1 out-of-fold predictions without refitting. The HR-unknown patient and three lesions were excluded only after prediction aggregation.

The HR source was the first primary-breast-tumor IHC assessment at initial diagnosis performed by the Department of Pathology, West China Hospital. No intracranial receptor IHC re-evaluation was available for this analysis. The original HR-unknown patient remained coded as missing for model analysis.

Repeat or salvage GKRS after the index treatment was not used for baseline radiomic extraction. If an index lesion progressed and subsequently underwent salvage GKRS, the event date was the first MRI showing local progression, or the salvage GKRS date when the radiographic date was unavailable. New brain metastases treated after index GKRS were considered distant intracranial events rather than local failure of the original lesion.

**2. Treatment-file and dose audit**

RTSTRUCT, RTPLAN and RTDOSE files were reviewed for readability, spatial consistency and lesion-to-target correspondence before exploratory dosimetric modeling. Treatment planning files were readable in all 109 reviewed folders, and MRI-to-RTSTRUCT as well as MRI-to-RTDOSE frame-of-reference consistency was confirmed in all reviewed folders. Because RT target names were inconsistent across cases, lesion-to-target correspondence was assessed by rasterizing RTSTRUCT target contours on the planning MRI grid and comparing them with the manual tumor ROI using spatial overlap, centroid distance, manual ROI volume, RT target volume and manual-review information. The treatment-file and dose-audit summary is provided in Supplementary Table S2.

Among 155 audited manual T1 tumor ROIs, 146 had comparable radiotherapy targets. Of these, 118 were classified as good/same, 3 as approximate and requiring review, and 25 as different or unmatched; 9 had no comparable RT target. Dose extraction yielded 159 dose-audit rows with successful or reviewable dose information. The exploratory dose-model subset contained 156 rows, including 122 endpoint-evaluable records with 34 events, and was not identical to the locked 105-lesion primary radiomics cohort. Lesions with approximate or unmatched targets were not automatically assigned target-level prescription variables unless mapping was manually accepted.

Extracted dose variables included treatment-session number, target volume, prescription or margin dose, prescription isodose line, minimum/mean/maximum dose, D98, D95, D50, D02, V12Gy, V100, V90, V50, D95 percent prescription, mean and maximum percent prescription, BED10 mean and maximum dose, EQD2_10 mean and maximum dose, equivalent single-fraction dose (α/β = 10 Gy) and equivalent single-fraction isodose line. Normal-tissue constraints were reviewed during clinical plan approval but were not used as locked predictors because critical-structure dose variables were not consistently extractable for all lesions.

For derived tumor-dose summaries, the linear-quadratic model was used to estimate biologically effective dose (BED). For an n-session GKRS course, total BED was calculated as BEDtotal = Σ[di × (1 + di/(α/β))], for i = 1 to n, where di denotes the prescription or margin dose of the i-th session. Tumor-related dose summaries used α/β = 10 Gy and were reported as BED10. The equivalent single-fraction dose was calculated by solving BEDtotal = Deq × (1 + Deq/(α/β)), giving Deq = [sqrt((α/β)^2 + 4 × (α/β) × BEDtotal) − (α/β)] / 2. For planned non-single-session GKRS, sufficient repair of sublethal damage between sessions was assumed, and no incomplete-repair correction was applied.

**3. Radiomics extraction, preprocessing and ET Rad-score construction**

The original extraction workflow was reported as using PyRadiomics version 3.1.0a2 with a fixed parameter file. Settings included bin width 25, normalization with normalizeScale 100, removal of outliers at 3 standard deviations, B-spline interpolation, 1-mm isotropic resampling, minimum ROI dimensionality 2 and minimum ROI size 10 voxels. The initial extraction framework covered Original, Wavelet and Laplacian-of-Gaussian images with sigma values of 1, 2 and 3 mm and an initial 1,130-feature matrix (107 Original, 744 Wavelet and 279 LoG features). This initial matrix must be distinguished from the locked primary-model input: 93 Original first-order and texture features, excluding shape features. The added reproducibility evaluation was limited to these 93 inputs and did not assess the full initial feature set. Its separately recorded software environment is described in **Supplementary Methods 5**.

All feature filtering, imputation, scaling, supervised ranking, redundancy filtering and coefficient estimation were restricted to training folds within patient-grouped repeated nested cross-validation. Candidate features with more than 25% missingness, near-zero variance or high redundancy were removed; remaining candidates were ranked within the training fold, standardized and fitted using ridge logistic regression with balanced class weights. Features were not selected using held-out fold outcomes. **Supplementary Table S4** reports fold-wise selection frequency, coefficient direction and final-fit membership separately.

Exploratory WT, non-enhancing tumor (NET), edema and Ring3 analyses were used to define the model boundary. Ring3 was generated as a 3-mm shell around the WT boundary and underwent automatic quality control and boundary-perturbation checks. Ring3 was retained as an exploratory compartment and was not included in the locked primary ET-HR model.

**4. Public-data support and claim boundaries**

Public datasets were used as tiered support rather than endpoint-matched validation. Dataset-specific support roles, analyses and claim boundaries are summarized in Supplementary Table S8.

BCBM-RadioGenomics was used for HR-related ET radiomic construct support by comparing HR-positive and HR-negative ET Rad-score and key ET feature distributions using Mann-Whitney U tests, Cliff's delta and Benjamini-Hochberg false-discovery-rate correction. PROTEAS and OpenBTAI/MOLAB were evaluated for endpoint-adjacent or technical support when imaging, masks and follow-up structure permitted. Domain-shift analyses summarized extraction success, missingness, outlier rates and feature-scale differences.

No public dataset contained the full combination of BCBM, GKRS/SRS treatment, comparable ET ROI, HR status and 12-month lesion-level local progression. Therefore, public data were used to support the HR-related radiomic construct and the external claim boundary, not to externally validate endpoint performance of the locked M1 model.

**5. Revision-stage inter-reader reproducibility**

Reproducibility was evaluated in the locked 49-patient, 105-lesion cohort using the two independent reader-specific segmentation sets retained before adjudication. Two neurosurgeons independently delineated the lesions. A senior neurosurgeon reviewed both sets of segmentations and adjudicated discrepancies. If disagreement persisted, the three neurosurgeons discussed the case and selected the final segmentation by majority opinion. Dice and ICC were calculated from these pre-adjudication sets; the final adjudicated segmentation was used for the primary model. The reproducibility evaluation was added during revision; no ICC threshold was used for original feature filtering and this reproducibility analysis did not alter the original feature set or locked primary model.

Dice similarity was computed on paired binary masks. For NET, both-present lesions were summarized separately from lesions with NET present in either reader. Single-reader absence was assigned Dice zero; both-reader absence was excluded from the Dice summary rather than assigned perfect agreement. HD95 was the 95th percentile of pooled bidirectional surface distances in physical units and was unavailable for single-empty pairs. Three NET-presence disagreements within the locked cohort were retained.

Feature extraction used PyRadiomics 3.1.0, SimpleITK 2.3.1, Python 3.9.13 and NumPy 1.26.4, with identical settings for both readers. Original-image features were enabled without shape features, using the original normalization, discretization and resampling settings. Image-mask geometry was checked with absolute tolerance 1e-5 and automatic mask correction was disabled. The reader-specific ET analysis masks were generated by the recorded processing script from each reader's manual segmentations. The final 93-feature inputs were assessed without model scaling, imputation or feature selection.

Single-measurement, absolute-agreement ICC(A,1) was calculated using the two-way ANOVA formula numerically corresponding to ICC(2,1). Patients were sampled with replacement 5,000 times, retaining all paired lesions for each sampled patient; the 2.5th and 97.5th percentiles defined the 95% confidence intervals. Intervals are conditional on the two readers. ICC point estimates of at least 0.750 or 0.900 are descriptive summaries, not retrospective feature-selection criteria. Complete feature results are provided in **Tables S9-S10**.

**6. Out-of-fold evaluation and resampling uncertainty**

The locked candidate pool comprised 93 Original non-shape ET features. Within each training split, features with more than 25% missingness were excluded, remaining missing values were median-imputed, and variance filtering used a 1e-10 threshold. Candidates were ranked by absolute outcome Spearman correlation, the top 50 were considered, and redundant features with absolute Spearman correlation of at least 0.90 were removed. Features were standardized before balanced ridge logistic regression (C = 0.1). At most the five largest absolute coefficients were retained; all other coefficients were set to zero without refitting, and the fitted intercept was retained.

The ET Rad-score was calculated as:

$$R_{i}=a_{0}+\sum_{j\in J} a_{j}z_{ij}$$

Where $z_{ij}$is the median-imputed radiomic feature standardized using the training-set mean and scaling factor, $J$ is the set of up to five retained features, $a_{j}$is the corresponding fitted coefficient, and $a_{0}$ is the fitted intercept. The score was not transformed into a probability before entering the final model.

HR status was coded as $H_{i}=0$ for HR-negative and $H_{i}=1$ for HR-positive disease. The product variable was calculated as $Q_{i}=R_{i}H_{i}$. For missing HR status, both $H_{i}$ and $Q_{i}$ were initially missing and were subsequently imputed using their respective training-column medians; the product was not recomputed after imputation. The three columns were then standardized separately using the training data. The final model was:

$$\mathrm{logit}(p_{i})=b_{0}+b_{R}Z_{Ri}+b_{H}Z_{Hi}+b_{Q}Z_{Qi}$$

where $p_{i}$ is the modelled probability of 12-month lesion-level local progression, and $Z_{Ri}$, $Z_{Hi}$ and $Z_{Qi}$ are the separately standardized score, HR and product variables. The reported standardized interaction coefficient is $b_{Q}$. For lesions with known HR status, a positive $b_{Q}$ indicates a greater increase in the modelled log odds of progression with increasing Rad-score in the HR-positive group than in the HR-negative group. The final model used balanced L2 logistic regression, with C selected by inner-fold mean Brier score from 0.01, 0.05, 0.1, 0.5, 1 and 2.

The primary lesion-level estimates use the mean of 20 raw outer-test probabilities per lesion. For exploratory calibration comparisons, calibrators were fitted to in-sample probabilities in each outer training set and applied to its outer test set; calibrator fitting was not based on inner out-of-fold probabilities. The primary model retained uncalibrated probabilities. Calibration curves and the slope/intercept estimated from aggregated out-of-fold predictions describe predictive calibration and did not transform primary predictions.

Sensitivity-targeted thresholds used the lower training-event probability quantile at 0.10 or 0.05; the specificity-targeted threshold used the higher training-non-event quantile at 0.90. These training thresholds were applied to held-out lesions in each repeat. For these targeted strategies, at least 11 positive classifications among each lesion's 20 held-out test classifications defined positivity; a 10:10 tie was classified as negative. Their reported performance therefore does not apply the median training threshold to the averaged probability. Targets refer to training operating points, not guaranteed test sensitivity or specificity. For descriptive median-risk stratification, each lesion's 20 OOF probabilities were first averaged, and the overall median across the 105 lesions (0.43956342220446454) was used as the cutoff. Mean probabilities at or above this cutoff defined high risk (53 lesions: 27 progressed and 26 non-progressed); lower values defined low risk (52 lesions: 6 progressed and 46 non-progressed). This grouping did not use training-fold median thresholds or majority voting. The OR and log-Wald 95% confidence interval were calculated after adding 0.5 to each of the four table cells, without covariate adjustment; the interval did not account for within-patient lesion clustering. Median and tertile risk groups based on averaged OOF probabilities are descriptive, not externally validated clinical rules.

For HR-specific uncertainty, 5,000 bootstrap resamples were drawn at patient level separately within each known-HR group, retaining all lesions per sampled patient. The 95% percentile confidence intervals are conditional on the saved predictions and do not incorporate uncertainty from retraining the full analytical pipeline. The HR-unknown patient contributed three lesions and one event and was excluded only from HR-specific summaries. Interaction coefficients were summarized over 100 correlated outer folds; their quartiles and range measure resampling stability rather than a population-effect confidence interval.

Average precision (AP) denotes the non-trapezoidal average-precision summary throughout; it is not used interchangeably with trapezoidal PR-AUC. For the reproducibility, HR-stratified and sensitivity bootstrap analyses described in Supplementary Methods 5, 6 and 8, the random seed was 20260920, except for the HR-positive subgroup analysis, which used 20260921. **Supplementary Tables S4 and S11-S12** provide feature-selection stability, subgroup uncertainty and interaction summaries.

**7. Revision-stage systemic treatment inventory**

The treatment inventory was linked by patient identifier only to the original locked cohort of 49 patients, grouped by any-lesion progression (22 progressed; 27 non-progressed). The descriptive window extended from index GKRS through the calendar 12-month anniversary, including both boundary dates. Regimens started before GKRS contributed their overlapping post-GKRS exposure. Regimen start and recorded end dates were retained; an end date representing an observation cutoff for ongoing treatment was not interpreted as verified treatment cessation. Confirmed treatment-free intervals were retained.

The verified GKRS-to-assessment MRI interval had a median of 385 days (range, 365-414); all 49 assessment scans occurred on or after the calendar 12-month anniversary. The patient-level indicator of ever receiving each treatment category was identical when the window ended at the anniversary or at the assessment MRI. Tables S13-S14 use the calendar-year window. This window check did not redefine or relabel the locked local-progression endpoint.

Drug aliases were normalized before classification: DS-8201 and T-DXd denote trastuzumab deruxtecan, and confirmed H entries denote trastuzumab. A name-only change was not counted as a drug switch. Regimens were defined by their recorded drug composition, not inferred doses, lines or intracranial efficacy. Antibody-drug conjugates were separated from conventional chemotherapy, and bone-directed supportive agents were identified separately. Unspecified aromatase-inhibitor treatment or ovarian suppression was retained at that level without assigning an unrecorded drug.

Table S13 counts each patient once per category and Table S14 once per distinct regimen received during the calendar-year window. Patients may contribute to several rows, so percentages need not sum to 100%. All 49 patients had a reconciled inventory. Two recorded treatment-free intervals do not indicate two patients untreated throughout follow-up. Baseline exposure was defined by a regimen start date on or before GKRS and a recorded end date on or after GKRS. One patient whose first regimen began 15 days after GKRS was confirmed to have received no preceding systemic treatment and was coded as unexposed at baseline. Whole-window ever-use was not entered as a baseline covariate because regimen changes could follow progression; baseline treatment sensitivity analyses are described in **Supplementary Methods 8**.

**8. Targeted clinical and baseline treatment sensitivity analyses**

Six revision-stage extensions were defined before inspecting their model results. Each added one covariate to ET Rad-score, HR and ET Rad-score × HR: natural-log baseline ET volume (cm³), prior WBRT, prior craniotomy, equivalent single-fraction dose (Gy; α/β = 10 Gy), HER2-TKI use active at GKRS or HER2-ADC use active at GKRS. The cohort and locked outcome labels remained 49 patients, 105 lesions and 33 events. WBRT and craniotomy histories were available for all patients (12 and 13 positive, respectively) and were examined separately rather than treating WBRT as a complete proxy for prior local treatment. Baseline HER2-TKI and HER2-ADC use were recorded in 23 and eight patients, respectively. Dose information for two lesions in one patient was flagged as unusable in the original data and was treated as missing only in the dose sensitivity analysis.

The same 20 repeats and five patient-grouped outer folds were reconstructed from the archived primary analysis. Within every outer training set, the ET Rad-score was refitted using the original 93-feature candidate pool and filtering procedure. The primary ET-HR model was also refitted as a reproducibility check: its maximum absolute difference from archived outer-test probabilities was less than 4 × 10⁻¹³. Each extension used training-fold median imputation, standardization and balanced L2 logistic regression. The original inner-selected C for that outer fold was retained without retuning for the extension. Thus the comparisons assess sensitivity conditional on the primary model's regularization, not the best achievable performance of newly optimized models. No held-out outcome was used for feature selection, imputation, scaling or fitting.

For each lesion, the 20 raw outer-test probabilities were averaged before calculating ROC-AUC, AP and Brier score. We drew 5,000 paired patient-cluster bootstrap samples, retaining all lesions from each sampled patient and using the same sampled patients for every model. Percentile 95% intervals were calculated for performance and paired differences from the reproduced primary model. These intervals condition on fixed predictions and do not include full-pipeline retraining uncertainty. Standardized interaction coefficients, their signs and paired changes from the primary-model coefficient were summarized descriptively across 100 correlated outer folds. Fold quartiles and ranges are stability summaries, not population-effect confidence intervals. No multiplicity-adjusted confirmatory tests were performed.

**Supplementary Table S15** gives model performance and fixed-prediction bootstrap intervals, Table S16 gives interaction-coefficient distributions, and Table S17 gives paired performance differences. All six extensions are reported. The predominant interaction direction was retained, but predictive performance did not consistently improve. The HER2-TKI extension had lower AP and higher Brier score, with paired intervals excluding zero; the dose extension also had a higher Brier score. These conditional exploratory findings do not estimate causal treatment effects. Whole-year treatment use was not substituted for baseline exposure, and no time-dependent treatment model was fitted because the regimen inventory had not been linked to lesion-specific first-progression dates.

**Supplementary Figures**

**Supplementary Figure S1.** Exploratory analyses and model boundaries. **(A)** Selected model comparisons across radiomic and clinical extensions. **(B)** Incremental-value stability across paired outer folds; Brier non-worse indicates no increase in Brier score relative to the comparator model. **(C)** Molecular sensitivity analyses. **(D)** Component retention decision summary. **(E)** Ring3 boundary perturbation analysis; Dilation +1 and Erosion −1 indicate one-step boundary perturbation in the implemented Ring3 robustness analysis. **(F)** Subgroup sensitivity analyses. These exploratory analyses show that WT, NET, Ring3, molecular subtype, HER2, Ki-67, inflammatory markers, lesion location and dosimetric variables did not provide sufficiently stable incremental value to replace the locked ET-HR interaction model. Ring3 was retained as exploratory rather than as a locked primary component.

**Supplementary Figure S2**. Tiered public-data support and external construct assessment. **(A)** Public datasets used for tiered support, showing availability of CE-T1 imaging, tumour masks, follow-up, HR labels, endpoint-adjacent information and HR-construct assessment. BCBM refers to the BCBM-RadioGenomics public dataset; Lim. indicates limited availability or limited endpoint suitability. **(B)** BCBM HR-related feature pattern showing Cliff’s delta for ET Rad-score and selected ET radiomic features, oriented as HR-positive minus HR-negative. **(C)** Endpoint-adjacent proxy assessment in PROTEAS and OpenBTAI/MOLAB, showing positive, negative and not-evaluable units across 6-month, 12-month and maximum follow-up windows. **(D)** Evidence tier and claim boundary, indicating HR-radiomic construct support from BCBM-RadioGenomics, limited endpoint-adjacent support from PROTEAS and limited or not-estimable endpoint-adjacent support from OpenBTAI/MOLAB. No complete endpoint-matched external validation of the locked M1 model is claimed.

**Supplementary Figure S3.** ROI, treatment-file and registration quality-control audit. **(A)** ROI audit counts across the source ROI list, T1 tumour ROI, manually delineated ET ROI, NET ROI and FLAIR abnormality region. QC audit denominators refer to the broader source/audit dataset and are not identical to the locked 105-lesion primary cohort. **(B)** Treatment/imaging audit showing successful DICOM-to-NRRD conversion, RTSTRUCT, RTPLAN and RTDOSE readability, and MRI/RT frame consistency. **(C)** Patient-level registration quality control for exploratory location-related analyses.

**Supplementary Figure S4.** Outer-fold performance variability. Distribution of outer-fold ROC-AUC, AP, Brier score, sensitivity and specificity across patient-grouped repeated nested cross-validation folds. This figure summarizes fold-level variability and complements the lesion-level out-of-fold performance estimates shown in the main text.

**Supplementary Figure S5.** Feature-scale domain shift relative to the local cohort. Heatmap summarizing feature-scale domain differences between the local cohort and public datasets for ET Rad-score and selected ET radiomic features. Large values indicate substantial feature-scale differences and support the decision not to claim complete endpoint-matched external validation. Missing or non-evaluable dataset-feature combinations are shown as not available.

**Supplementary Table**

**Supplementary Table S1. Cohort flow and HR-status completeness.**

| **Level** | **Category** | **n** | **Notes** |
| --- | --- | --- | --- |
| Patient | Screened for eligibility | 61 | Standardized clinical, GKRS lesion and radiomics records |
| Patient | Included in final analysis cohort | 49 | Locked primary cohort |
| Lesion | Screened for eligibility | 159 | Standardized lesion-level radiomics records |
| Lesion | Included in locked lesion-level cohort | 105 | 33 progressed and 72 non-progressed lesions |
| HR status | Known HR status | 48/49 patients; 102/105 lesions | Used for HR-stratified analyses |
| HR status | Unknown HR status | 1/49 patients; 3/105 lesions | Handled by training-fold median imputation in M1 |
| Lesion | Excluded before model development | 54 | Non-overlapping primary reasons below |
| Lesion | Radiologically adjudicated radiation necrosis | 9 | Clinical imaging adjudication, not pathological confirmation |
| Lesion | Death within 12 months without 12-month imaging | 24 | Primary exclusion reason |
| Lesion | Incomplete imaging follow-up | 1 | Primary exclusion reason |
| Lesion | Lesions excluded solely by pretreatment WT volume | 20 | Pretreatment WT volume <0.064 cm³ or >10 cm³; excludes lesions already assigned to the preceding categories. |
| Patient | No eligible lesion remaining | 12 | Patient exclusion follows lesion-level eligibility; not the sum of patients across overlapping reasons |

Note: Each excluded lesion was assigned one primary exclusion reason. Six lesions excluded because of radiation necrosis or inadequate follow-up also fell outside the pretreatment WT volume range of 0.064–10 cm³ and were not counted again among the 20 lesions excluded solely on the basis of volume.

**Supplementary Table S2. GKRS treatment-file and dose-audit summary.**

| **Audit item** | **Result** | **Role** |
| --- | --- | --- |
| RT objects readable | RTSTRUCT/RTPLAN/RTDOSE readable in 109/109 folders | Treatment-file audit |
| Frame-of-reference consistency | Confirmed in 109/109 folders | Spatial consistency audit |
| Audited manual T1 tumour ROIs | 155 | Target-matching denominator |
| Comparable RT targets | 146/155 | Target-match denominator |
| Good/same target match | 118/146 | Spatially concordant match |
| Approximate target match | 3/146 | Audit review only unless manually accepted |
| Different or unmatched target | 25/146 | Broader treatment-file review |
| Dose-model subset | 156 rows; 122 endpoint-evaluable records; 34 events | Exploratory dosimetric modelling |

Note: Denominators refer to the broader treatment-file audit and are not identical to the locked 105-lesion primary radiomics cohort.

**Supplementary Table S3. Radiomics extraction and modelling settings.**

| **Item** | **Setting** |
| --- | --- |
| Primary ROI | Manual pretreatment ET ROI on contrast-enhanced T1-weighted MRI |
| Preprocessing | N4 correction, 1-mm isotropic resampling, intensity standardization |
| Image types | Broader extraction: Original, Wavelet and LoG. Locked primary model: Original only. |
| Feature classes | Locked primary model: 93 first-order and texture features, excluding shape. |
| ET Rad-score construction | Training-fold imputation, filtering, ranking, redundancy removal, standardization and ridge logistic regression |
| Primary model | ET Rad-score + HR status + ET Rad-score × HR status |
| Validation | Patient-grouped repeated nested cross-validation: 20 repeats, 5 outer folds, 4 inner folds |

Note: All data-dependent steps were restricted to training folds. Abbreviations: ET, enhancing tumour; HR, hormone receptor; LoG, Laplacian of Gaussian.

**Supplementary Table S4. Outer-fold ET feature-selection frequency and coefficient direction.**

| Feature (ET_original_ omitted) | Selected /100 (%) | Mean coefficient* | Positive / negative* | Final model |
| --- | --- | --- | --- | --- |
| glszm_GrayLevelVariance | 74 (74%) | 0.280 | 74 / 0 | Yes |
| firstorder_Maximum | 68 (68%) | 0.255 | 68 / 0 | Yes |
| glszm_LargeAreaHighGrayLevelEmphasis | 52 (52%) | 0.274 | 52 / 0 | Yes |
| glcm_Correlation | 46 (46%) | 0.231 | 46 / 0 | No |
| glszm_ZoneVariance | 39 (39%) | 0.261 | 39 / 0 | No |
| glszm_HighGrayLevelZoneEmphasis | 36 (36%) | 0.265 | 36 / 0 | No |
| glcm_MCC | 33 (33%) | 0.233 | 33 / 0 | No |
| glcm_Imc2 | 29 (29%) | 0.253 | 29 / 0 | No |
| glrlm_RunVariance | 28 (28%) | 0.300 | 28 / 0 | Yes |
| glszm_ZonePercentage | 18 (18%) | -0.245 | 0 / 18 | No |
| glcm_Idmn | 16 (16%) | 0.227 | 16 / 0 | Yes |
| gldm_SmallDependenceLowGrayLevelEmphasis | 9 (9%) | -0.198 | 0 / 9 | No |
| glszm_SizeZoneNonUniformityNormalized | 9 (9%) | -0.276 | 0 / 9 | No |
| glszm_GrayLevelNonUniformityNormalized | 6 (6%) | 0.280 | 6 / 0 | No |
| firstorder_Kurtosis | 5 (5%) | 0.290 | 5 / 0 | No |
| glszm_LowGrayLevelZoneEmphasis | 5 (5%) | -0.221 | 0 / 5 | No |
| glcm_ClusterProminence | 4 (4%) | 0.227 | 4 / 0 | No |
| gldm_GrayLevelNonUniformity | 4 (4%) | 0.206 | 4 / 0 | No |
| glszm_LargeAreaEmphasis | 4 (4%) | 0.290 | 4 / 0 | No |
| glcm_ClusterShade | 3 (3%) | 0.293 | 3 / 0 | No |
| firstorder_Range | 2 (2%) | 0.161 | 2 / 0 | No |
| firstorder_Skewness | 2 (2%) | 0.242 | 2 / 0 | No |
| glcm_Imc1 | 2 (2%) | -0.266 | 0 / 2 | No |
| glcm_Idn | 1 (1%) | 0.150 | 1 / 0 | No |
| gldm_DependenceEntropy | 1 (1%) | -0.240 | 0 / 1 | No |
| gldm_LargeDependenceHighGrayLevelEmphasis | 1 (1%) | 0.147 | 1 / 0 | No |
| ngtdm_Busyness | 1 (1%) | 0.263 | 1 / 0 | No |
| ngtdm_Contrast | 1 (1%) | -0.182 | 0 / 1 | No |
| ngtdm_Strength | 1 (1%) | 0.233 | 1 / 0 | No |

Note: Features selected in at least one of 100 outer training folds are shown. Frequency is the selection count divided by 100. Feature selection frequency across outer folds is a descriptive measure of resampling stability and does not represent a formal variable selection probability in a single fixed model. *Mean coefficients and direction counts are conditional on selection and use standardized features. Final-model membership identifies the five features in the final fitted ET Rad-score and is distinct from selection frequency. Selection used training-fold data only. ET, enhancing tumor; GLCM, gray-level co-occurrence matrix; GLRLM, gray-level run-length matrix; GLSZM, gray-level size-zone matrix; NGTDM, neighboring gray-tone difference matrix.

**Supplementary Table S5. Performance of the primary ET-HR interaction model.**

| **Analysis** | **Evaluation unit** | **ROC-AUC (95% CI)** | **AP (95% CI)** | **Brier score (95% CI)** |
| --- | --- | --- | --- | --- |
| Lesion-equal primary analysis | 105 lesions | 0.750 (0.642-0.851) | 0.567 (0.402-0.768) | 0.204 (0.177-0.235) |
| Patient-balanced analysis | 105 lesions, inverse patient lesion-count weighting | 0.786 (0.671-0.885) | 0.621 (0.431-0.837) | 0.194 (0.158-0.235) |
| Patient-level maximum lesion risk | 49 patients | 0.803 (0.664-0.922) | 0.694 (0.508-0.914) | 0.200 (0.161-0.244) |

Note: Metrics were calculated from held-out out-of-fold predictions. Patient-balanced metrics used inverse patient lesion-count weighting. Patient-level maximum risk refers to the highest predicted lesion risk per patient. Lower Brier scores indicate better probabilistic performance. AP, average precision; ROC-AUC, receiver operating characteristic area under the curve.

**Supplementary Table S6. HR-known restricted evaluation using locked M1 out-of-fold predictions.**

| **Evaluation set** | **Denominator** | **ROC-AUC** | **AP** | **Brier score** |
| --- | --- | --- | --- | --- |
| HR-known restricted set | 48 patients / 102 lesions | 0.755 | 0.573 | 0.202 |
| Full primary prediction set | 49 patients / 105 lesions | 0.750 | 0.567 | 0.204 |

Note: The model was not refitted; the HR-unknown patient and three lesions were excluded after prediction aggregation.

**Supplementary Table S7. Threshold-based clinical utility analyses.**

| **Strategy** | **Sensitivity** | **Specificity** | **PPV** | **NPV** | **Risk fraction** | **Additional result** |
| --- | --- | --- | --- | --- | --- | --- |
| 90%-target low-risk exclusion | 0.848 | 0.472 | 0.424 | 0.872 | Low-risk 37.1% | Progressions below threshold: 5/33 |
| 95%-target low-risk exclusion | 0.879 | 0.319 | 0.372 | 0.852 | Low-risk 25.7% | Sensitivity analysis; progressions below threshold: 4/33 |
| 90%-target high-risk confirmation | 0.485 | 0.875 | 0.640 | 0.788 | High-risk 23.8% | Progressions not flagged high-risk: 17/33 |
| Median-risk stratification | 0.818 | 0.639 | 0.509 | 0.885 | High risk: 53; low risk: 52 | Progressed/non-progressed: high risk 27/26; low risk 6/46. Progression rates 50.9% vs 11.5%; OR 7.42 (95% CI, 2.79–19.76). |
| Patient-level maximum risk | - | - | - | - | 49 patients | ROC-AUC 0.803; AP 0.694; Brier 0.200 |

Note: Targeted strategies used training-fold thresholds and at least 11 positive votes among 20 held-out classifications per lesion; 10:10 ties were negative. Descriptive median-risk groups used the overall median of mean OOF probabilities (approximately 0.4396; unrounded cutoff used): values at or above the cutoff defined high risk (n = 53), and lower values defined low risk (n = 52). The OR used a 0.5 addition to each table cell and a log-Wald 95% CI, without covariate adjustment or adjustment for within-patient lesion clustering. AP, average precision; NPV, negative predictive value; PPV, positive predictive value.

**Supplementary Table S8. Public-data support roles and analysis framework.**

| **Dataset / analysis** | **Support role** | **Analysis performed** | **Claim boundary** |
| --- | --- | --- | --- |
| BCBM-RadioGenomics | Construct support | Mann-Whitney U test, Cliff's delta and FDR correction | HR-associated ET construct patterns; not endpoint validation |
| PROTEAS | Endpoint-adjacent support | Proxy endpoint assessment when evaluable | Limited by sample and proxy endpoint |
| OpenBTAI/MOLAB | Endpoint-adjacent or technical support | Endpoint availability and event-structure assessment | Discrimination not estimated when endpoint structure was insufficient |
| Domain-shift assessment | Technical support | Extraction success, missingness, outlier rate and feature-scale differences | Technical compatibility and claim boundary |

Note: Public datasets were not treated as complete endpoint-matched external validation of the locked M1 model.

**Supplementary Table S9. Inter-reader segmentation agreement.**

| Region / population | Lesions | Dice median (IQR) | HD95 mm median (IQR) |
| --- | --- | --- | --- |
| WT | 105 | 0.949 (0.911-0.966) | 0.700 (0.469-1.170) |
| ET | 105 | 0.937 (0.894-0.958) | 0.718 (0.469-1.170) |
| NET, both present | 48 | 0.910 (0.838-0.955) | 0.681 (0.552-1.231) |
| NET, present in either | 51 | 0.905 (0.822-0.954) | 48 evaluable pairs |

Note: WT and ET include 49 patients. NET both-present and either-present subsets include 33 and 34 patients, respectively. NET was absent for both readers in 54 lesions, and present for only one reader in 3 lesions. The two NET summaries overlap and must not be added together.

**Supplementary Table S10. Inter-reader ICCs for the 93 ET candidate features.**

| Feature (ET_original_ prefix omitted) | ICC | 95% interval | Final model |
| --- | --- | --- | --- |
| firstorder_10Percentile | 0.859 | 0.715-0.941 | No |
| firstorder_90Percentile | 0.989 | 0.972-0.998 | No |
| firstorder_Energy | 0.999 | 0.998-1.000 | No |
| firstorder_Entropy | 0.947 | 0.902-0.972 | No |
| firstorder_InterquartileRange | 0.919 | 0.838-0.965 | No |
| firstorder_Kurtosis | 0.876 | 0.729-0.943 | No |
| firstorder_Maximum | 0.992 | 0.973-1.000 | Yes |
| firstorder_MeanAbsoluteDeviation | 0.916 | 0.824-0.965 | No |
| firstorder_Mean | 0.967 | 0.941-0.982 | No |
| firstorder_Median | 0.970 | 0.946-0.984 | No |
| firstorder_Minimum | 0.761 | 0.633-0.856 | No |
| firstorder_Range | 0.877 | 0.797-0.932 | No |
| firstorder_RobustMeanAbsoluteDeviation | 0.913 | 0.815-0.966 | No |
| firstorder_RootMeanSquared | 0.977 | 0.959-0.987 | No |
| firstorder_Skewness | 0.956 | 0.924-0.976 | No |
| firstorder_TotalEnergy | 0.999 | 0.998-1.000 | No |
| firstorder_Uniformity | 0.957 | 0.908-0.980 | No |
| firstorder_Variance | 0.901 | 0.789-0.961 | No |
| glcm_Autocorrelation | 0.814 | 0.710-0.895 | No |
| glcm_ClusterProminence | 0.908 | 0.714-0.972 | No |
| glcm_ClusterShade | 0.862 | 0.700-0.963 | No |
| glcm_ClusterTendency | 0.910 | 0.809-0.961 | No |
| glcm_Contrast | 0.951 | 0.898-0.980 | No |
| glcm_Correlation | 0.879 | 0.802-0.927 | No |
| glcm_DifferenceAverage | 0.963 | 0.925-0.983 | No |
| glcm_DifferenceEntropy | 0.962 | 0.923-0.981 | No |
| glcm_DifferenceVariance | 0.940 | 0.862-0.979 | No |
| glcm_Id | 0.974 | 0.950-0.986 | No |
| glcm_Idm | 0.975 | 0.952-0.986 | No |
| glcm_Idmn | 0.904 | 0.853-0.941 | Yes |
| glcm_Idn | 0.928 | 0.890-0.955 | No |
| glcm_Imc1 | 0.895 | 0.793-0.947 | No |
| glcm_Imc2 | 0.905 | 0.826-0.951 | No |
| glcm_InverseVariance | 0.950 | 0.916-0.969 | No |
| glcm_JointAverage | 0.865 | 0.790-0.922 | No |
| glcm_JointEnergy | 0.964 | 0.909-0.985 | No |
| glcm_JointEntropy | 0.955 | 0.915-0.976 | No |
| glcm_MCC | 0.803 | 0.679-0.880 | No |
| glcm_MaximumProbability | 0.961 | 0.895-0.988 | No |
| glcm_SumAverage | 0.865 | 0.790-0.922 | No |
| glcm_SumEntropy | 0.939 | 0.888-0.968 | No |
| glcm_SumSquares | 0.922 | 0.835-0.966 | No |
| glrlm_GrayLevelNonUniformity | 0.996 | 0.993-0.998 | No |
| glrlm_GrayLevelNonUniformityNormalized | 0.955 | 0.909-0.976 | No |
| glrlm_GrayLevelVariance | 0.901 | 0.789-0.961 | No |
| glrlm_HighGrayLevelRunEmphasis | 0.814 | 0.709-0.897 | No |
| glrlm_LongRunEmphasis | 0.966 | 0.940-0.986 | No |
| glrlm_LongRunHighGrayLevelEmphasis | 0.847 | 0.759-0.915 | No |
| glrlm_LongRunLowGrayLevelEmphasis | 0.905 | 0.820-0.968 | No |
| glrlm_LowGrayLevelRunEmphasis | 0.871 | 0.748-0.942 | No |
| glrlm_RunEntropy | 0.945 | 0.908-0.965 | No |
| glrlm_RunLengthNonUniformity | 0.990 | 0.978-0.996 | No |
| glrlm_RunLengthNonUniformityNormalized | 0.957 | 0.926-0.979 | No |
| glrlm_RunPercentage | 0.966 | 0.941-0.983 | No |
| glrlm_RunVariance | 0.974 | 0.954-0.989 | Yes |
| glrlm_ShortRunEmphasis | 0.951 | 0.915-0.979 | No |
| glrlm_ShortRunHighGrayLevelEmphasis | 0.811 | 0.703-0.893 | No |
| glrlm_ShortRunLowGrayLevelEmphasis | 0.852 | 0.729-0.933 | No |
| glszm_GrayLevelNonUniformity | 0.993 | 0.985-0.996 | No |
| glszm_GrayLevelNonUniformityNormalized | 0.905 | 0.880-0.930 | No |
| glszm_GrayLevelVariance | 0.890 | 0.812-0.938 | Yes |
| glszm_HighGrayLevelZoneEmphasis | 0.767 | 0.634-0.866 | No |
| glszm_LargeAreaEmphasis | 0.977 | 0.952-0.989 | No |
| glszm_LargeAreaHighGrayLevelEmphasis | 0.954 | 0.918-0.983 | Yes |
| glszm_LargeAreaLowGrayLevelEmphasis | 0.942 | 0.857-0.963 | No |
| glszm_LowGrayLevelZoneEmphasis | 0.815 | 0.692-0.899 | No |
| glszm_SizeZoneNonUniformity | 0.981 | 0.968-0.987 | No |
| glszm_SizeZoneNonUniformityNormalized | 0.867 | 0.791-0.912 | No |
| glszm_SmallAreaEmphasis | 0.888 | 0.820-0.930 | No |
| glszm_SmallAreaHighGrayLevelEmphasis | 0.792 | 0.649-0.891 | No |
| glszm_SmallAreaLowGrayLevelEmphasis | 0.757 | 0.606-0.849 | No |
| glszm_ZoneEntropy | 0.951 | 0.920-0.969 | No |
| glszm_ZonePercentage | 0.947 | 0.905-0.970 | No |
| glszm_ZoneVariance | 0.979 | 0.957-0.991 | No |
| gldm_DependenceEntropy | 0.951 | 0.914-0.973 | No |
| gldm_DependenceNonUniformity | 0.990 | 0.978-0.996 | No |
| gldm_DependenceNonUniformityNormalized | 0.974 | 0.954-0.985 | No |
| gldm_DependenceVariance | 0.977 | 0.957-0.990 | No |
| gldm_GrayLevelNonUniformity | 0.996 | 0.993-0.998 | No |
| gldm_GrayLevelVariance | 0.903 | 0.794-0.961 | No |
| gldm_HighGrayLevelEmphasis | 0.820 | 0.716-0.900 | No |
| gldm_LargeDependenceEmphasis | 0.964 | 0.936-0.986 | No |
| gldm_LargeDependenceHighGrayLevelEmphasis | 0.886 | 0.813-0.945 | No |
| gldm_LargeDependenceLowGrayLevelEmphasis | 0.898 | 0.864-0.974 | No |
| gldm_LowGrayLevelEmphasis | 0.874 | 0.758-0.944 | No |
| gldm_SmallDependenceEmphasis | 0.938 | 0.895-0.963 | No |
| gldm_SmallDependenceHighGrayLevelEmphasis | 0.831 | 0.716-0.907 | No |
| gldm_SmallDependenceLowGrayLevelEmphasis | 0.794 | 0.636-0.902 | No |
| ngtdm_Busyness | 0.915 | 0.865-0.954 | No |
| ngtdm_Coarseness | 0.953 | 0.915-0.978 | No |
| ngtdm_Complexity | 0.893 | 0.805-0.944 | No |
| ngtdm_Contrast | 0.912 | 0.806-0.969 | No |
| ngtdm_Strength | 0.898 | 0.827-0.941 | No |

Note: Estimates use 105 paired lesions in 49 patients. Absolute-agreement, single-measurement ICCs have percentile 95% intervals from 5,000 patient-cluster bootstrap resamples. Median ICC was 0.928 (range, 0.757-0.999); all 93 point estimates were at least 0.750, and 62 were at least 0.900. Lower 95% limits were at least 0.750 for 75 features. Final-model membership differs from outer-fold selection frequency.

**Supplementary Table S11. HR-stratified performance with patient-cluster 95% confidence intervals.**

| Group | Patients / lesions / events | ROC-AUC (95% CI) | AP (95% CI) | Brier (95% CI) |
| --- | --- | --- | --- | --- |
| HR-negative | 30 / 70 / 21 | 0.702 (0.554-0.831) | 0.562 (0.360-0.776) | 0.215 (0.183-0.245) |
| HR-positive | 18 / 32 / 11 | 0.827 (0.614-1.000) | 0.664 (0.409-1.000) | 0.174 (0.119-0.249) |

Note: Events are progressed lesions. Thirteen HR-negative and eight HR-positive patients had at least one progressed lesion. Each interval uses 5,000 valid patient-cluster bootstrap resamples with fixed out-of-fold predictions. The HR-unknown patient (three lesions, one event) is omitted here, not from primary modeling.

**Supplementary Table S12. Standardized ET Rad-score × HR coefficients across 100 outer folds.**

| Median | Q1-Q3 | Minimum-maximum | Positive / zero / negative |
| --- | --- | --- | --- |
| 0.326 | 0.207-0.445 | -0.087 to 1.154 | 98 / 0 / 2 |

Note: The primary lesion-equal weighting scheme is shown. These correlated outer-fold estimates describe direction and spread under repeated resampling; their quantiles are not confidence limits for an independent population interaction effect.

**Supplementary Table S13. Systemic treatment categories during the 12-month post-GKRS window.**

| Category | Progressed n=22 | Non-progressed n=27 | All n=49 |
| --- | --- | --- | --- |
| CDK4/6 inhibitor | 0 (0.0) | 1 (3.7) | 1 (2.0) |
| HER2-targeted ADC | 4 (18.2) | 10 (37.0) | 14 (28.6) |
| HER2 tyrosine kinase inhibitor | 11 (50.0) | 14 (51.9) | 25 (51.0) |
| HER2-targeted monoclonal antibody | 5 (22.7) | 5 (18.5) | 10 (20.4) |
| PARP inhibitor | 1 (4.5) | 1 (3.7) | 2 (4.1) |
| Antiangiogenic therapy | 0 (0.0) | 4 (14.8) | 4 (8.2) |
| Bone-directed supportive therapy | 0 (0.0) | 1 (3.7) | 1 (2.0) |
| Conventional chemotherapy | 19 (86.4) | 20 (74.1) | 39 (79.6) |
| Endocrine therapy / ovarian suppression | 2 (9.1) | 5 (18.5) | 7 (14.3) |
| Immune checkpoint inhibitor | 0 (0.0) | 1 (3.7) | 1 (2.0) |
| Other ADC | 1 (4.5) | 4 (14.8) | 5 (10.2) |
| Other targeted therapy | 0 (0.0) | 2 (7.4) | 2 (4.1) |

Note: Values are patients, n (%). Progressed denotes at least one progressed lesion. Categories overlap; ADCs are not counted as conventional chemotherapy. Bone-directed agents are supportive rather than intracranial anticancer treatment. The inventory includes combinations and sequential treatment, potentially after progression, and is not a baseline exposure comparison. ADC, antibody-drug conjugate; CDK4/6, cyclin-dependent kinase 4/6; PARP, poly(ADP-ribose) polymerase.

**Supplementary Table S14. Recorded systemic regimens during the 12-month post-GKRS window by patient progression group.**

| Recorded drugs / treatment strategy | Progressed n=22 | Non-progressed n=27 | Total |
| --- | --- | --- | --- |
| Capecitabine + Pyrotinib | 7 (31.8) | 8 (29.6) | 15 |
| Capecitabine | 6 (27.3) | 4 (14.8) | 10 |
| Trastuzumab deruxtecan | 4 (18.2) | 3 (11.1) | 7 |
| Pyrotinib | 2 (9.1) | 3 (11.1) | 5 |
| Sacituzumab govitecan | 1 (4.5) | 3 (11.1) | 4 |
| Trastuzumab emtansine | 1 (4.5) | 3 (11.1) | 4 |
| Olaparib | 1 (4.5) | 1 (3.7) | 2 |
| Pyrotinib + Vinorelbine | 1 (4.5) | 1 (3.7) | 2 |
| Tamoxifen | 1 (4.5) | 1 (3.7) | 2 |
| Vinorelbine | 1 (4.5) | 1 (3.7) | 2 |
| Abemaciclib + Fulvestrant + Goserelin | 0 (0.0) | 1 (3.7) | 1 |
| Alpelisib + Eribulin | 0 (0.0) | 1 (3.7) | 1 |
| Anastrozole + Bevacizumab + Capecitabine + Eribulin + Ovarian suppression (unspecified) | 0 (0.0) | 1 (3.7) | 1 |
| Anastrozole + Ovarian suppression (unspecified) + Trastuzumab deruxtecan | 0 (0.0) | 1 (3.7) | 1 |
| Anlotinib | 0 (0.0) | 1 (3.7) | 1 |
| Apatinib + Camrelizumab + Trastuzumab deruxtecan | 0 (0.0) | 1 (3.7) | 1 |
| Aromatase inhibitor (unspecified) + Ovarian suppression (unspecified) + Pyrotinib + Trastuzumab | 0 (0.0) | 1 (3.7) | 1 |
| Aromatase inhibitor (unspecified) + Ovarian suppression (unspecified) + Pyrotinib + Trastuzumab + Zoledronic acid | 0 (0.0) | 1 (3.7) | 1 |
| Bevacizumab + Capecitabine + Eribulin | 0 (0.0) | 1 (3.7) | 1 |
| Bevacizumab + Etoposide + Utidelone | 0 (0.0) | 1 (3.7) | 1 |
| Bevacizumab + Trastuzumab deruxtecan | 0 (0.0) | 1 (3.7) | 1 |
| Capecitabine + Carmustine + Pyrotinib + Trastuzumab | 1 (4.5) | 0 (0.0) | 1 |
| Capecitabine + Pyrotinib + Temozolomide + Trastuzumab | 1 (4.5) | 0 (0.0) | 1 |
| Capecitabine + Pyrotinib + Trastuzumab | 0 (0.0) | 1 (3.7) | 1 |
| Capecitabine + Pyrotinib + Trastuzumab deruxtecan | 1 (4.5) | 0 (0.0) | 1 |
| Capecitabine + Trastuzumab | 1 (4.5) | 0 (0.0) | 1 |
| Capecitabine + Trastuzumab deruxtecan + Vinorelbine | 0 (0.0) | 1 (3.7) | 1 |
| Carboplatin + Eribulin | 1 (4.5) | 0 (0.0) | 1 |
| Denosumab + Letrozole + Pyrotinib | 0 (0.0) | 1 (3.7) | 1 |
| Disitamab vedotin | 0 (0.0) | 1 (3.7) | 1 |
| Disitamab vedotin + Neratinib + Pembrolizumab | 0 (0.0) | 1 (3.7) | 1 |
| Epirubicin + Nab-paclitaxel | 0 (0.0) | 1 (3.7) | 1 |
| Eribulin + Pertuzumab + Trastuzumab | 0 (0.0) | 1 (3.7) | 1 |
| Etoposide | 1 (4.5) | 0 (0.0) | 1 |
| Etoposide + Pyrotinib | 1 (4.5) | 0 (0.0) | 1 |
| Everolimus + Letrozole | 0 (0.0) | 1 (3.7) | 1 |
| Exemestane + Pyrotinib | 1 (4.5) | 0 (0.0) | 1 |
| Gemcitabine + Neratinib + Trastuzumab emtansine | 0 (0.0) | 1 (3.7) | 1 |
| Gemcitabine + Sacituzumab govitecan | 0 (0.0) | 1 (3.7) | 1 |
| Inetetamab + Pyrotinib + Vinorelbine | 1 (4.5) | 0 (0.0) | 1 |
| Inetetamab + Tucatinib + Vinorelbine | 0 (0.0) | 1 (3.7) | 1 |
| Inetetamab + Vinorelbine | 1 (4.5) | 0 (0.0) | 1 |
| Pyrotinib + S-1 + Trastuzumab | 0 (0.0) | 1 (3.7) | 1 |
| Pyrotinib + Trastuzumab | 1 (4.5) | 0 (0.0) | 1 |
| Pyrotinib + Trastuzumab + Vinorelbine | 0 (0.0) | 1 (3.7) | 1 |
| Pyrotinib + Trastuzumab deruxtecan | 0 (0.0) | 1 (3.7) | 1 |
| Thiotepa + Utidelone | 1 (4.5) | 0 (0.0) | 1 |
| Utidelone | 0 (0.0) | 1 (3.7) | 1 |

Note: Each patient is counted once per distinct drug combination and may appear in multiple rows. Combinations include recorded bone-directed agents. DS-8201 and T-DXd denote trastuzumab deruxtecan; S-1 denotes tegafur/gimeracil/oteracil. The window ends at the calendar 12-month anniversary, not the assessment MRI. These descriptive distributions may include treatment initiated after progression and do not indicate treatment line or efficacy (Supplementary Methods 7).

**Supplementary Table S15. Targeted sensitivity analysis performance with patient-cluster 95% intervals.**

| **Model** | **ROC-AUC (95% CI)** | **AP (95% CI)** | **Brier (95% CI)** |
| --- | --- | --- | --- |
| Primary ET-HR model | 0.750 (0.645-0.846) | 0.567 (0.398-0.762) | 0.204 (0.175-0.235) |
| + Log ET volume | 0.745 (0.645-0.838) | 0.549 (0.388-0.744) | 0.205 (0.174-0.236) |
| + Prior WBRT | 0.734 (0.624-0.837) | 0.542 (0.360-0.739) | 0.210 (0.176-0.243) |
| + Prior craniotomy | 0.758 (0.654-0.853) | 0.584 (0.443-0.761) | 0.195 (0.161-0.230) |
| + Equivalent single-fraction dose | 0.730 (0.625-0.835) | 0.547 (0.383-0.743) | 0.209 (0.179-0.240) |
| + Baseline HER2-TKI | 0.741 (0.637-0.838) | 0.545 (0.380-0.740) | 0.207 (0.177-0.238) |
| + Baseline HER2-ADC | 0.736 (0.622-0.838) | 0.571 (0.387-0.756) | 0.207 (0.176-0.240) |

Note: All models include 49 patients, 105 lesions and 33 events. Each extension adds one covariate and retains the primary model’s fold-specific regularization parameter C. Estimates use each lesion’s mean of 20 held-out probabilities. Intervals use 5,000 paired patient-cluster bootstrap resamples with fixed predictions. Original primary-model intervals are reported separately in Table S5. TKI, tyrosine kinase inhibitor; ADC, antibody-drug conjugate; WBRT, whole-brain radiotherapy.

**Supplementary Table S16. ET Rad-score × HR coefficient stability in targeted sensitivity analyses.**

| **Model** | **Median (Q1-Q3)** | **Minimum to maximum** | **Positive / negative folds** | **Paired change median (Q1-Q3)** |
| --- | --- | --- | --- | --- |
| Primary ET-HR model | 0.326 (0.207-0.445) | -0.087 to 1.154 | 98 / 2 | +0.000 (-0.000 to +0.000) |
| + Log ET volume | 0.314 (0.197-0.444) | -0.108 to 1.156 | 98 / 2 | -0.007 (-0.014 to -0.003) |
| + Prior WBRT | 0.325 (0.208-0.437) | -0.103 to 1.132 | 97 / 3 | -0.000 (-0.005 to +0.001) |
| + Prior craniotomy | 0.332 (0.212-0.484) | -0.027 to 1.174 | 98 / 2 | +0.002 (-0.009 to +0.019) |
| + Equivalent single-fraction dose | 0.324 (0.205-0.446) | -0.084 to 1.235 | 98 / 2 | -0.000 (-0.006 to +0.004) |
| + Baseline HER2-TKI | 0.325 (0.209-0.446) | -0.090 to 1.202 | 98 / 2 | -0.000 (-0.004 to +0.004) |
| + Baseline HER2-ADC | 0.321 (0.202-0.442) | -0.090 to 1.141 | 98 / 2 | -0.004 (-0.013 to +0.000) |

Note: Standardized coefficients are from balanced, penalized logistic regression; none were zero. Paired change is the extension coefficient minus the primary-model coefficient in the same outer fold. Quartiles and ranges describe stability across 100 correlated folds, not population-effect confidence intervals.

**Supplementary Table S17. Paired performance differences from the primary ET-HR model.**

| **Added covariate** | **ΔROC-AUC (95% CI)** | **ΔAP (95% CI)** | **ΔBrier (95% CI)** |
| --- | --- | --- | --- |
| Log ET volume | -0.005 (-0.021 to +0.009) | -0.018 (-0.053 to +0.008) | +0.001 (-0.003 to +0.005) |
| Prior WBRT | -0.017 (-0.052 to +0.014) | -0.025 (-0.089 to +0.026) | +0.005 (-0.003 to +0.015) |
| Prior craniotomy | +0.008 (-0.063 to +0.069) | +0.017 (-0.090 to +0.117) | -0.009 (-0.028 to +0.013) |
| Equivalent single-fraction dose | -0.021 (-0.055 to +0.001) | -0.019 (-0.053 to +0.005) | +0.005 (0.000 to +0.012) |
| Baseline HER2-TKI | -0.010 (-0.021 to 0.000) | -0.022 (-0.046 to -0.004) | +0.003 (+0.002 to +0.005) |
| Baseline HER2-ADC | -0.015 (-0.049 to +0.012) | +0.004 (-0.055 to +0.046) | +0.003 (-0.005 to +0.011) |

Note: Differences are extension minus primary model. Higher ROC-AUC/AP and lower Brier score are preferable. Intervals use the same 5,000 paired patient-cluster resamples across models, with predictions fixed and no bootstrap refitting. Comparisons retain the primary model’s fold-specific regularization parameters.
